# Nitidine Chloride Alleviates Inflammation and Cellular Senescence in Murine Osteoarthritis Through Scavenging ROS

**DOI:** 10.3389/fphar.2022.919940

**Published:** 2022-07-22

**Authors:** Changjian Lin, Lujie Ge, Luping Tang, Yuzhe He, Safwat Adel Abdo Moqbel, Kai Xu, Diana Ma, Xing Zhou, Jisheng Ran, Lidong Wu

**Affiliations:** ^1^ Department of Orthopedic Surgery, The Second Affiliated Hospital, Zhejiang University School of Medicine, Hangzhou, China; ^2^ Orthopedics Research Institute of Zhejiang University, Hangzhou, China; ^3^ Key Laboratory of Motor System Disease Research and Precision Therapy of Zhejiang Province, Hangzhou, China; ^4^ Clinical Research Center of Motor System Disease of Zhejiang Province, Hangzhou, China; ^5^ Department of Emergency Medicine, the Second Affiliated Hospital, Zhejiang University School of Medicine, Hangzhou, China

**Keywords:** osteoarthritis, nitidine chloride, ROS, senescence, NLRP3 inflammasome, MAPK, NF-κB

## Abstract

Osteoarthritis (OA) is one of the most common chronic musculoskeletal disorder worldwide, representing a major source of disability, pain and socioeconomic burden. Yet the effective pharmaceutical treatments applied in the clinical works are merely symptomatic management with uncertainty around their long-term safety and efficacy, namely no drugs currently are capable of modulating the biological progression of OA. Here, we identified the potent anti-inflammatory as well as anti-oxidative properties of Nitidine Chloride (NitC), a bioactive phytochemical alkaloid extracted from natural herbs, in IL-1β-treated rat articular chondrocytes (RACs), LPS-stimulated RAW 264.7 and rat osteoarthritic models *in vivo*. We demonstrated NitC remarkably inhibited the production of inflammatory mediators including COX2 and iNOS, suppressed the activation of MAPK and NF-κB cell signaling pathway and reduced the expression of extracellular matrix (ECM) degrading enzymes including MMP3, MMP9 and MMP13 in IL-1β-treated RACs. Several emerging bioinformatics tools were performed to predict the underlying mechanism, the result of which indicated the potential reactive oxygen species (ROS) clearance potential of NitC. Further, NitC exhibited its anti-oxidative potential through ameliorating cellular senescence in IL-1β-treated RACs and decreasing NLRP3 inflammasomes activation in LPS-stimulated RAW 264.7 *via* scavenging ROS. Additionally, X-ray, micro-CT and other experiments *in vivo* demonstrated that intra-articular injection of NitC significantly alleviated the cartilage erosion, ECM degradation and subchondral alterations in OA progression. In conclusion, the present study reported the potent anti-inflammatory and anti-oxidative potential of NitC in OA biological process, providing a promising therapeutic agent for OA management.

## Introduction

Osteoarthritis (OA) is a debilitating disease with an unmet medical need, imposing substantial and increasing burdens on the socioeconomic budget. For currently 240 million affected individuals worldwide, this already suffering musculoskeletal disorder is becoming more burdensome when its specific treatment has been limited to end-stage surgery (Hunter and Bierma-Zeinstra, 2019; Katz et al., 2021). Asides from education and constructed exercise, effective non-surgical interventions are confined to topical or oral analgesics for short-term pain relief (Jones et al., 2019; Latourte et al., 2020). The serious challenge urges further research to facilitate identifications of emerging pharmaceutical therapies to slow down the biological process of OA progression and eventually reduce the burdens.

Accumulating evidence indicated that OA is a multifaceted condition, associated with low-grade chronic inflammation, extracellular matrix degradation, oxidative stress, chondrocytes cellular senescence as well as other alterations in articular and periarticular tissues (Liu-Bryan and Terkeltaub, 2015; Robinson et al., 2016; Mobasheri et al., 2017; Coryell et al., 2021). Activation of mitogen-activated protein kinases (MAPK) and nuclear factor κB (NF-κB) cell signaling pathway is a hallmark in OA pathophysiology (Ran et al., 2018; Moqbel et al., 2020). The regulating mechanisms underlying abundant phytomedicine or small molecule studies associated with OA treatment rely on exactly inhibiting MAPK or NF-κB pathway (Guo et al., 2022; Yeo et al., 2022). Furthermore, imbalance in reactive oxygen species (ROS) generation and clearance is another leading cause of oxidative damage, redox-modulated cell signaling pathway disruption and cartilage degradation (Bai et al., 2019; Bolduc et al., 2019; Park et al., 2021; Kang et al., 2022). Liang et al. (2020) reported intra-articular injection of nanofibrous membranes to eliminate ROS significantly attenuated OA progression. Jeon et al. (2017) demonstrated clearance of local senescent chondrocytes ameliorated the development of post-traumatic OA and created a pro-regenerative circumstance. All of these advances in the understanding of OA structural progression have pushed forward the discovery of promising pharmacological therapies, which may potentially reshape the landscape of OA management.

Nitidine Chloride (NitC), a bioactive phytochemical alkaloid extracted from the herb *Zanthoxylum nitidum*, has been reported to inhibit several enzymes from the cytochrome P450 super family (Li et al., 2014; Mao et al., 2018; Mao et al., 2019), manifesting potential anti-oxidative capacity. Meanwhile, research in the field indicated the potent anti-inflammatory property of NitC (Wang et al., 2012; Yang et al., 2019). Nevertheless, attention is mainly converged on the anticancer activity of NitC (Cui et al., 2020). The effect of NitC on inflammation and oxidative stress in OA progression has not been reported yet.

Therefore, the present study was undertaken to examine the impact of NitC on OA models established through IL-1β induction or under destabilization of the medial meniscus (DMM) surgery. Based on the substantial experiments, briefly, our results first report the anti-inflammatory and anti-oxidative properties of NitC on murine osteoarthritic models both *in vitro* and *in vivo* and its underlying mechanism, providing a brand-new drug choice for OA management.

## Materials and Methods

### Reagents

Nitidine Chloride (HY-N0498) and Menadione (HY-B0332) were purchased from MedChemExpress. Dulbecco’s Modified Eagle Medium (DMEM; catalog:11320033), fetal bovine serum (FBS), trypsin-EDTA (catalog: 25200072) and Penicillin-Streptomycin (catalog:15070063) were all purchased from Gibco, NY, USA. Recombinant rat IL-1β (501-RL-010/CF) was obtained from R&D Systems, Abingdon, UK. Collagenase II, DMSO and Lipopolysaccharide (LPS; catalog: L6529) were obtained from Sigma-Aldrich, Merck KGaA, MO, USA.

### Cells Culture

Rat articular chondrocytes isolation was conducted as previously described (Ma et al., 2018; Moqbel et al., 2020). In brief, bilateral hips harvested from 4-week-old Sprague Dawley rats (weighing 140 ± 20 g) were cut into 1 mm^3^ particle under the sterile circumstance. Then the cartilage was digested with 0.25% tyrosine for 30 min and subsequently mixed up with type II collagenase on a horizontal shaker at 37°C for another 4 h. The chondrocytes were isolated through centrifuging the cell suspension, followed by seeded into culture flasks with DMEM (10% FBS and 1% penicillin-streptomycin) at 37°C with 5% CO_2_. These colony chondrocytes were regarded as passage 0 (P0). Normal P1-3 chondrocytes were used in this study. Mouse macrophages cell line RAW 264.7 was purchased from the Cell Bank of Shanghai Institutes for Biological Science of the Chinese Academy of Sciences (TCM13, Shanghai, China). RAW 254.7 was cultured with DMEM (10% FBS and 1% penicillin-streptomycin) at 37°C with 5% CO_2._

### Chondrocytes Viability Analysis

CCK-8 assay (KGA317, KeyGen Biotech, Nanjing, China) was performed according to the manufacturer’s instruction to assess the cytotoxicity of NitC on chondrocytes. P3 chondrocytes were seeded into 96-well plates for 24 h under normal circumstances as mentioned above. Then the culture media was respectively substituted for 0, 1, 10, 25, and 50 μM NitC for 24 and 48 h. After that, the media was replaced with fresh DMEM media containing 10% CCK-8 solution for further 3 h incubation at 37°C. The optical density (OD) was determined and the chondrocytes viability was calculated then. This experiment was repeated three times.

### Quantitative Real-Time Polymerase Chain Reaction (qRT-PCR)

Total RNA was extracted using the TRIzol^®^ Plus RNA Purification Kit (Invitrogen, Carlsbad, CA, United States). The RNA concentrations were detected and adjusted before reverse transcription with PrimeScript™ RT Master Mix (RR036A, Takara, Beijing, China). cDNA samples were replicated with SYBR^®^ Premix Ex Taq™ II (RR82WR, Takara, Beijing, China) with an Applied Biosystems™ 7500 Fast Real-Time PCR System (Applied Biosystems, Foster City, CA, United States). 18S was used as an endogenous control. The primers used are listed in Supplementary Table S1. All of the above experiments were performed in triplicate according to the manufacturer’s instructions. The data were calculated using the 2^−ΔΔCT^ method.

### Safranin O and Alcian Blue Staining

To detect the chondrocyte extracellular matrix phenotype alterations, Safranin O and Alcian Blue staining assays were conducted in this study. P3 chondrocytes were seeded into 12-well plates for 24 h, followed by the intervention of different concentrations (0, 1, 10, 25, and 50 μM) of NitC for 24 h. Then the cells were fixed with 4% paraformaldehyde solution for 15 min. The fixed cells were washed with PBS three times and then stained with 0.1% safranin O solution for 5 min. As for Alcian Blue Staining, the fixed cells were stained with 1% Alcian Blue solution for 30 min. The stained cells were washed with PBS and then imaged by a gross camera and Lecia inverted optical microscope.

### β-Galactosidase Activity Assay

The senescence detected assay was conducted as previously described (Xu et al., 2021). Briefly, pretreated chondrocytes in 12-well plates were incubated with β-Galactosidase staining buffer for 24 h from β-Galactosidase activity assay (C0602, Beyotime Biotechnology, China) according to the manufacturer’s instructions. Then the cellular senescence was measured under Lecia inverted optical microscope.

### Reactive Oxygen Species Detection

The ROS detection was performed using Reactive Oxygen Species Assay Kit (S0033M, Beyotime Biotechnology, China) as previously described (Bao et al., 2021). For flow cytometry analysis, pretreated chondrocytes were cultured with DCFH-DA at 37°C for 30 min. After being washed three times with PBS, chondrocytes were harvested from 6-well plates and subsequently ROS was evaluated using Beckman CytoFLEX cytometer. For fluorescent analysis, pretreated chondrocytes were incubated with DCFH-DA in 12-well plates at 37°C for 30 min. After washing three times, fluorescent intensities were observed by the inverted Lecia Fluorescence Microscope.

### Bioinformatics Analysis

To further elucidate the underlying mechanism of NitC treatment, several emerging bioinformatics were used in the present study. In brief, PharmMapper was used to predict the potential proteins directly binding with NitC according to the instructions (Liu et al., 2010; Wang et al., 2016; Wang et al., 2017). Thereafter, database STRING was used to establish the protein-protein association network (Szklarczyk et al., 2019). Cytoscape was subsequently used to calculate the top 10 proteins by the MCC method in the network (Shannon et al., 2003; Otasek et al., 2019). The bioinformatics analysis results were shown in Supplementary Tables S2, S3.

### Western Blot

The adherent chondrocytes were washed three times with PBS and then detached with a cell scraper. Then the harvested cells were lysed in RIPA Lysis Buffer (P0013B, Beyotime, China) containing a protease inhibitor cocktail (P1005, Beyotime, China) as well as a protein phosphatase inhibitor (P1260, Solarbio Science & Technology, Beijing, China) on ice for 30 min. After extraction, proteins were separated using SDS-PAGE gels at 70–140 V for 1.5 h and transferred onto polyvinylidene difluoride (PVDF) membranes (IPVH00010, Millipore, United States) at 250 mA for 2 h. Subsequently, the membranes were blocked for 2 h with 5% BSA (A600903, Sangon Biotech, China) at room temperature. Then the membranes were incubated with primary antibody at 4°C overnight. The next day, after being washed by TBST 3 times, the membrane was incubated with horseradish peroxidase- (HRP-) conjugated goat anti-rabbit and anti-mouse secondary antibodies (A0208, A0216; 1:5,000, Beyotime, China) for 2 h and the luminescence was determined using an ECL kit (WBKLS005, Immobilon, KGaA). The relative amount of proteins was analyzed using ImageJ and normalized to GAPDH (GA) or β-Actin. All assays were performed in triplicate.

### Animal Model

Twenty-four male Sprague Dawley rats (200–250 g; 6 weeks old) were randomly divided into three groups (six rats/four cages per group): sham group (Sham), osteoarthritis group (DMM) and NitC treated group (DMM + NitC). Rats in the Sham group underwent sham surgery, while the other rats underwent Destabilization of the medial meniscus (DMM) surgery and were thereby regarded as OA rats (Xu et al., 2020). One week after Sham or DMM surgery, rats in group Sham and DMM were intra-articularly injected with vehicles while rats in group DMM + NitC were injected with an equal volume of 10 μM NitC. These weekly injections lasted 6 weeks. Then the rats were sacrificed 1 week after the last treatment, and knee samples were fixed with 4% paraformaldehyde solution for further research.

### Radiological Assessment

After fixation, the rat knee samples were scanned with an X-ray imager. The representative images in each group showed the alteration of articular surface and space width. As for micro-CT analysis, the samples were assessed using a U-CT system (MILabs U-CT, Netherlands). MILabs Rec 10.16 software was used to evaluate the data. Three-dimensional bone structure image slices and several trabecular bone indices including trabecular bone volume fraction (BV/TV; %), trabecular thickness (Tb.Th; cm) and trabecular spacing (Tb.Sp; cm) were calculated and reconstructed using the IMALYTICS Preclinical 2.1 software.

### Histological Analysis

The fixed knee joints were decalcified with 10% EDTA over 2 months. The specimens were dehydrated using ascending ethanol series and embedded in paraffin blocks and subsequently were cut into 5 μm sections. Then the sections of the joint were stained with SO and HE according to the manufacturers’ instructions. Immunofluorescence analysis of sections was performed subsequently.

### Immunofluorescence

For p65 detection in chondrocytes, pretreated chondrocytes were fixed with 4% paraformaldehyde for 10 min, followed by permeabilization with 0.1% v/v Triton X-100 for 5 min. After being washed by PBS 3 times, chondrocytes were incubated with primary antibody against p65 at 4°C overnight. On the next day, chondrocytes were incubated with fluorochrome-conjugated secondary antibodies for 2 h in the dark. Then the chondrocytes were stained with DAPI for 5 min and finally observed by inverted Lecia Fluorescence Microscope.

### Statistical Analysis

All the results in the present stud are presented as means ± SDs. For *in vitro* studies, One-way analysis of variance (ANOVA) followed by Tukey’s test was used for comparison between the two groups. As for *in vivo* studies, analysis for multiple samples was carried out by the one-way ANOVA with Tukey’s post-hoc test. Statistical analysis was performed with Prism 8 (IBM Corporation, Armonk, NY, United States). The value of *p* < 0.05 was considered indicative of significant statistical differences.

## Results

### Impacts of NitC on Primary RACs

**FIGURE 1:**
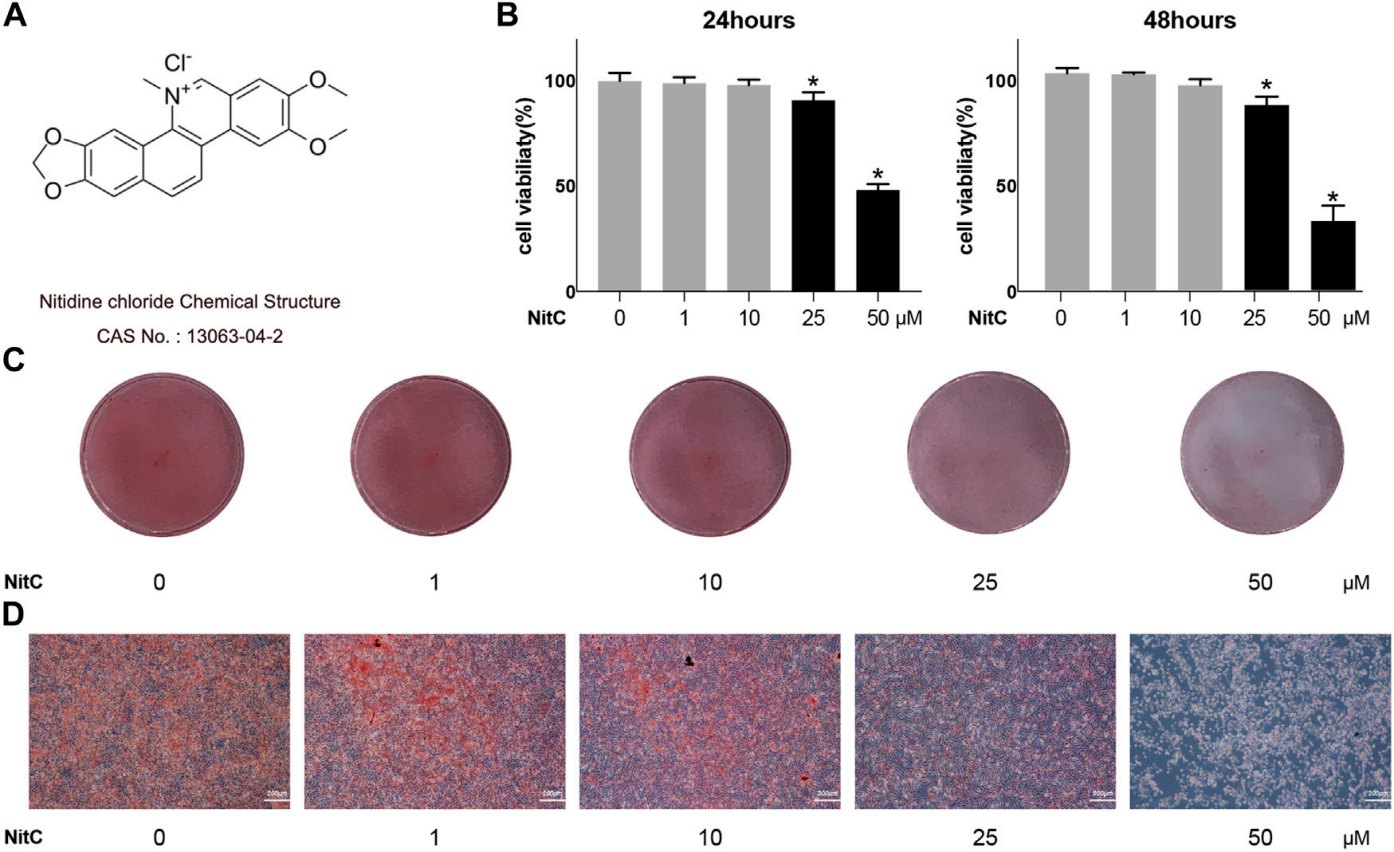
Effect of NitC on viability and chondrocyte phenotype maintenance. **(A)** Nitidine Chloride (NitC) chemical structure. **(B)** CCK8 analysis of NitC on chondrocytes viability. **(C)** Gross view of safranin O stained primary rat articular chondrocytes treated with 0, 1, 10, 25, or 50 μM NitC in 12 wells for 24 h. **(D)** Microscopic images of safranin O stained primary rat chondrocytes treated with 0, 1, 10, 25, or 50 μM NitC for 24 h. *p < 0.05 versus the control group. Scale bar = 200 μM.

The chemical structure of Nitidine Chloride (NitC) was shown in Figure 1A. To begin with, a CCK8 assay was conducted to figure out the appropriate concentration of NitC on primary articular rat chondrocytes (RACs). NitC did not a manifest detrimental effect till its concentration rose to 25 μM, both in 24- and 48-h treating time (Figure 1B). Aligned with the CCK8 assay, safranin O staining results revealed that when the NitC concentration excessed 10 μM, the application of NitC contributed to the extracellular matrix degradation (Figures 1C,D). Based on these findings, 1 and 10 μM concentrations of NitC were used for further *in vitro* tests.

### NitC Inhibited Inflammation Progress in RACs

**FIGURE 2:**
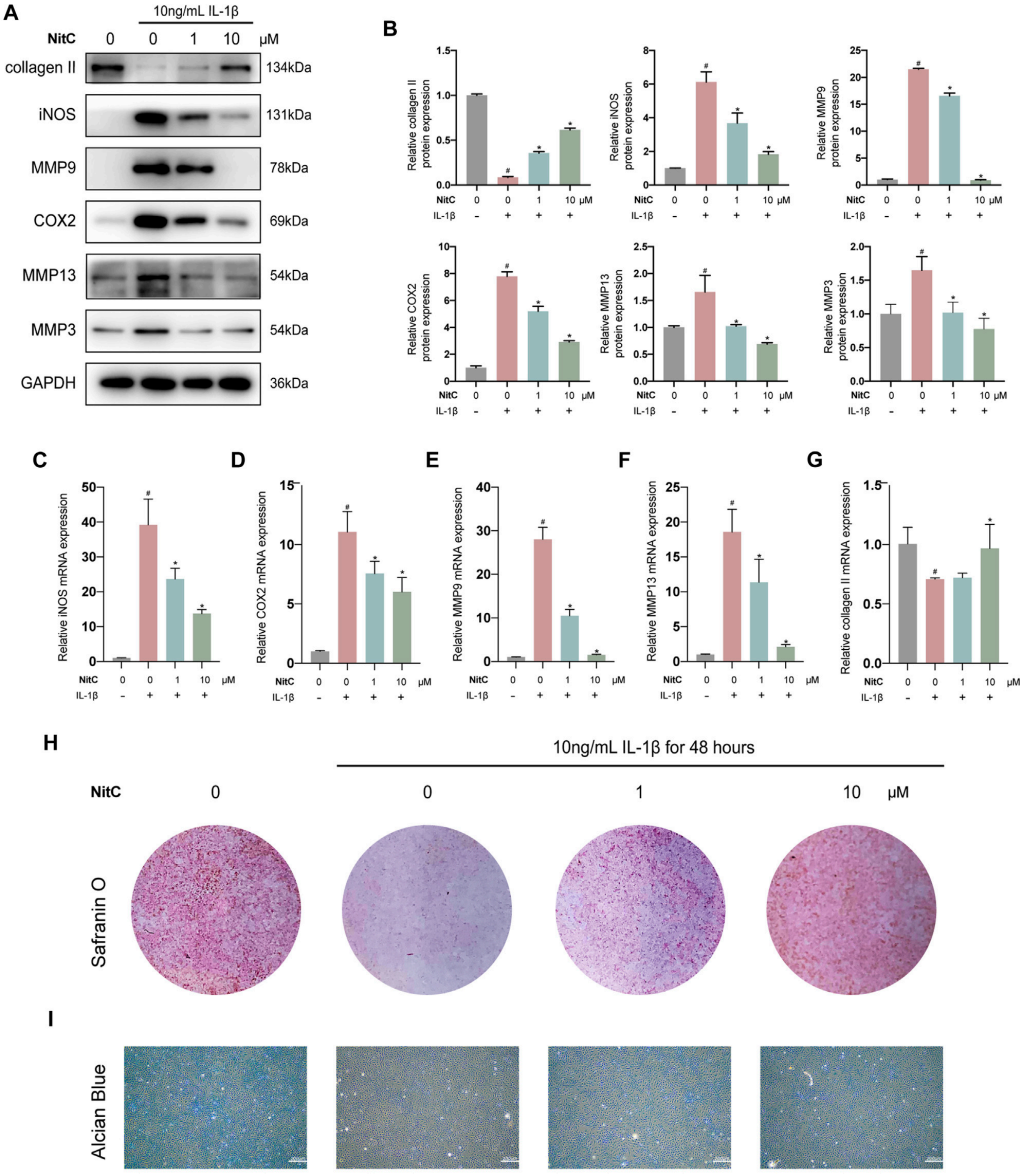
NitC alleviated ECM degradation and inhibited RACs inflammation *in vitro*. **(A)** The WB analysis of COL2, iNOS, MMP9, COX2, MMP13, MMP3, and GA was conducted and their relative protein expression **(B)** was shown. **(C–G)** The qRT-PCR analysis was performed to detect the relative mRNA expression of iNOS, MMP9, MMP13, COX2, and COL2. **(H)** Representative images of the Safranin O staining of pretreated chondrocytes. **(I)** The microscopic images of Alcian Blue staining of pretreated chondrocytes. *#p* < 0.05 versus the control group. ∗*p* < 0.05 versus the model group. Scale bars = 200 μM.

Recently, accumulating studies in humans and animal models demonstrated the crucial role of inflammation in osteoarthritis pathogenesis (Liu-Bryan and Terkeltaub, 2015; Robinson et al., 2016; Conaghan et al., 2019). Interleukin-1β (IL-1β)-induced osteoarthritis *in vitro* model has been widely recognized in the corresponding research field (Xu et al., 2020; Bao et al., 2021). Thus, we firstly investigated whether NitC was entitled with anti-inflammation capacity in IL-1β-treated RACs. Briefly, passage 3(P3) RACs were pretreated with different concentrations of NitC for 1 h, followed by incubation with 10 ng/ml IL-1β for 24 h. As shown in Figures 2A,B, NitC decreased the protein expression of universally acknowledged inflammation-related mediators including iNOS and COX2 in a dose-dependent manner. Meanwhile, qRT-PCR results in Figures 2C,D revealed that NitC could also down-regulate iNOS and COX2 expression at the mRNA level. These results indicated that NitC possessed potent anti-inflammatory ability through decreasing the secretion of inflammatory mediators.

### NitC Reduced Extracellular Matrix Degradation

The normal joint function requires the biomechanical structural support and resistance to deformation from the cartilage extracellular matrix, which is mainly composed of proteoglycans within the collagen fibrillar network (Krishnan and Grodzinsky, 2018). The early feature of initiation characteristic to OA is the upregulation of cartilage degrading enzymes including matrix metalloproteinase 3 (MMP3), MMP9, and MMP13 (Liu-Bryan and Terkeltaub, 2015; Mobasheri et al., 2017; Mehana et al., 2019). Therefore, we examined the effect of NitC on IL-1β-induced RACs. Western blot analysis in Figures 2A,B demonstrated that IL-1β treatment significantly enhanced the production of MMP3, MMP9, and MMP13, whereas the intervention of NitC remarkably rescued the enzymes over-production in a dose-dependent manner. Furthermore, the qRT-PCR analysis indicated that NitC treatment suppressed the MMP9 and MMP13 at the mRNA level (Figures 2E,F).

To further examine the alterations in NitC-treated RACs, Western blot and qRT-PCR analyses were performed to detect the expression of type II collagen. In accordance with the upregulations of MMPs, type II collagen decreased sharply in IL-1β-treated RACs both at proteins level and mRNA level, while the intervention of NitC significantly reversed it (Figures 2A,B,G). In addition, Safranin O staining as well as Alcian Blue staining results indicated that loss of cartilage matrix proteoglycan glycosaminoglycan (GAG) induced by IL-1β was restored by the treatment of NitC (Figure 2H). Taking these results together, we demonstrated that NitC was capable of downregulating MMP3 and enhancing GAG expression, ultimately decreasing ECM degradation in IL-1β-induced RACs.

### NitC Inhibited the IL-1β-Induced Activation of MAPK and NF-κB Pathway

**FIGURE 3:**
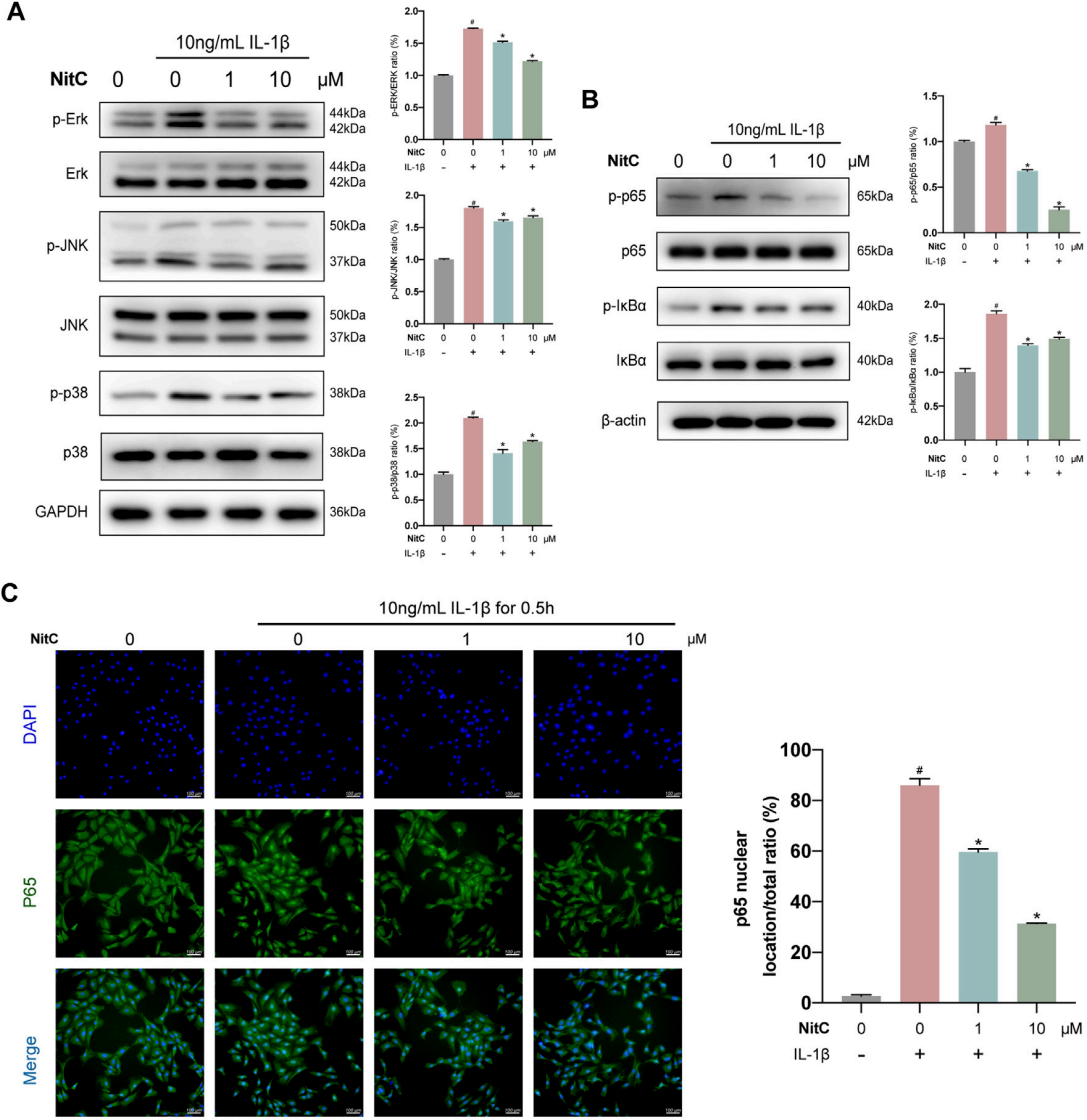
NitC inhibited MAPK and NF-κB pathway in the osteoarthritic model *in vitro*. **(A)** The WB analysis of MAPK pathway-related protein: p-ERK, ERK, p-JNK, JNK, p-p38, and p38 as well as endogenous control GA were performed as previously described. Besides, the p-ERK/ERK, p-JNK/JNK, and p-p38/p38 ratios were shown. **(B)** The WB analysis of NF-κB pathway-related protein: p-p65, p65, p-IκBα, IκBα, and endogenous control β-Actin were conducted. The p-p65/p65 and p-IκBα/IκBα ratios were shown. **(C)** The nuclear translocation of p65 was assessed by immunofluorescent microscopy. The quantified results of nuclear location/total ratio were shown as well. Blue: DAPI. Green: p65. *#p* < 0.05 versus the control group. ∗*p* < 0.05 versus the model group. Scale bars = 100 μM.

Activation of mitogen-activated protein kinases (MAPK) and nuclear factor κB (NF-κB) cell signaling pathway is another hallmark in OA pathophysiology (Ran et al., 2018; Moqbel et al., 2020). Our previous studies found that inhibited MAPK pathway or NF-κB pathway could significantly suppress the progression of OA (Ma et al., 2018; Ran et al., 2018). Here, we examined the effect of NitC on the MAPK pathway in IL-1β-induced RACs. Western blot analysis detected that IL-1β stimulation activated by increasing the expression of phosphorylation of Erk, JNK, and p38 levels, whereas NitC treatment could reverse it (Figure 3A). The activation status of p65 and IκBα was examined through Western blot analysis as well, and the results revealed that NitC treatment could remarkably rescue the activation of the NF-κB pathway in IL-1β-induced RACs (Figure 3B). Furthermore, immunofluorescence analysis was performed to detect the location of p65, the key effector of the NF-κB pathway, and the result demonstrated nuclear translocation of p65 was significantly blocked by pretreatment of NitC in a dose-dependent manner (Figure 3C). Based on these results, we identified that NitC was able to effectively inhibit the activation of MAPK and NF-κB cell signaling pathways in IL-1β pretreated RACs.

### NitC Was Capable of Inhibiting ROS Generation

**FIGURE 4:**
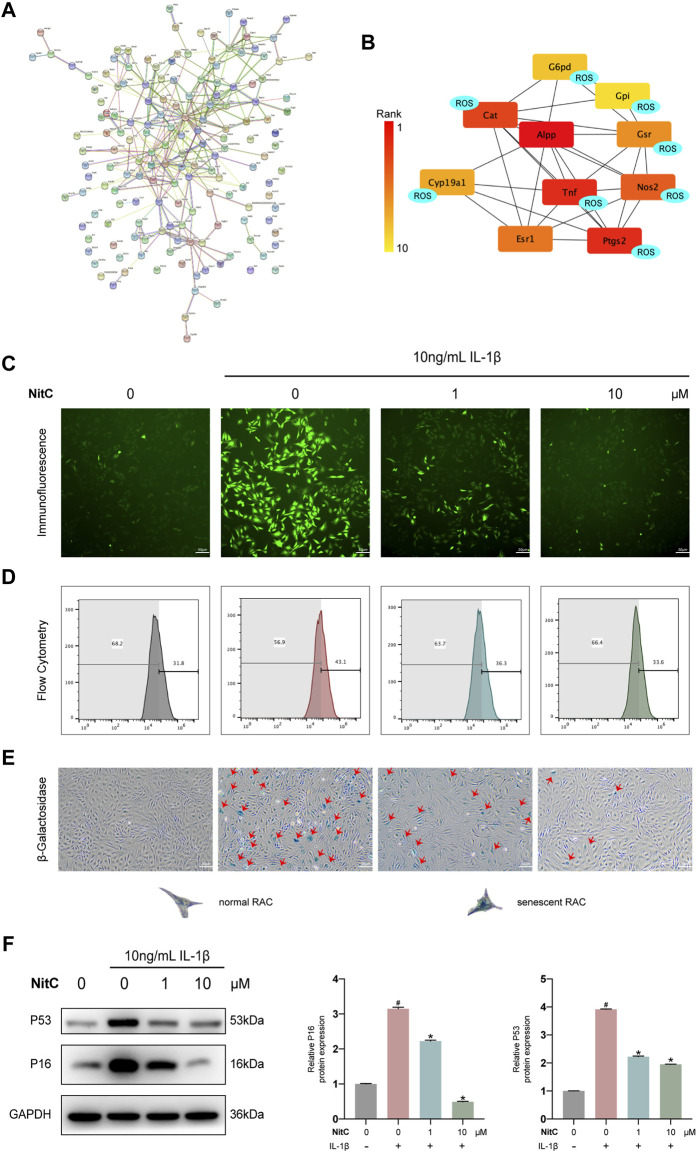
NitC reduced ROS generation and ameliorated cellular senescence in RACs. **(A)** The potential targets of NitC were obtained by PharmMapper. The protein-protein association network of the targets was established by STRING. **(B)** The top 10 hub genes were calculated by Cytoscape subsequently. Genes marked with “ROS” are involved with the modulation of ROS generation according to the existing research. **(C)** Immunofluorescent microscopy was utilized to detect the ROS in RACs. The intensity of fluorescence represented the relative level of ROS. **(D)** The ROS levels of different groups were also assessed through flow cytometry. **(E)** β-Galactosidase activity assay was performed in RACs. Typical normal RAC and senescent RAC were shown. Red arrows mark the senescent RAC. **(F)** The WB analysis of cellular senescence-related genes: p16 and p53 were conducted. Meanwhile, the quantified results of relative protein expression were present. *#p* < 0.05 versus the control group. ∗*p* < 0.05 versus the model group. Scale bars = 50 μM.

To further elucidate the underlying mechanism of NitC on RACs, several emerging bioinformatics were performed as our previous study (Xu et al., 2021). To begin with, PharmMapper was used to predict the potential protein targets directly binding with NitC. The top 300 candidates of potential proteins were listed in Supplementary Table S2. Database STRING was subsequently used to establish the protein-protein association network (Figure 4A). Meanwhile, software Cytoscape was used to calculate the top 10 proteins which played a crucial role in the network. Interestingly, the predictive results indicated eight proteins from the candidates were tightly involved with ROS generation or clearance (Figure 4B). For example, tumor necrosis factor (TNF), a regulator of the generation of ROS and reactive nitrogen species (RNS), and prostaglandin-endoperoxide synthase 2 (Ptgs2), encoded by COX2 and already investigated in the present study (Blaser et al., 2016; Chen et al., 2021). To probe the promising ROS scavenging property, immunofluorescence analysis and flow cytometry analysis were conducted. The results showed treated with IL-1β significantly enhanced ROS generation, while the intervention of NitC could decrease the level of ROS in a dose-dependent manner (Figures 4C,D). Combining bioinformatics prediction with substantial experiments, we revealed NitC was capable of reducing oxidative stress in IL-1β treated RACs *via* scavenging ROS.

### NitC Attenuated Cellular Senescence in IL-1β-Treated RACs

Although multiple risk factors such as obesity, trauma and heredity participate in OA pathophysiology, the most prevalent one is aging (O’Neill et al., 2018). Senescence, a hallmark in aging, is defined by a permanent state of cell arrest. Jeon et al. (2017) demonstrated clearance of local senescent chondrocytes ameliorated the development of post-traumatic OA and created a pro-regenerative circumstance. Advance in exploring the cellular senescence has facilitated a novel approach to modify the progression of OA. Recent findings supported that besides aging, cellular senescence could be induced by oxidative stress, including the excess level of ROS (Tudorachi et al., 2021). Therefore, we investigated the effect of NitC on chondrocyte cellular senescence. RACs were cultured on 6-well plates and pretreated with NitC for 1 h, followed by stimulation of IL-1β. Subsequently, a β-Galactosidase activity assay was conducted to measure the cellular senescence. The results showed that IL-1β stimulation remarkably arose the number of senescent chondrocytes, whereas the pretreatment of NitC relieved the effect (Figure 4E). In addition, Western blot analysis showed that NitC managed to downregulate the protein expression of p16 and p53, acknowledged biomarkers in the senescence process, induced by IL-1β. In conclusion, these results indicated that NitC was capable of ameliorating cellular senescence in IL-1β-induced RACs.

### Regeneration of ROS Rescued the Effect of NitC on RACs

**FIGURE 5:**
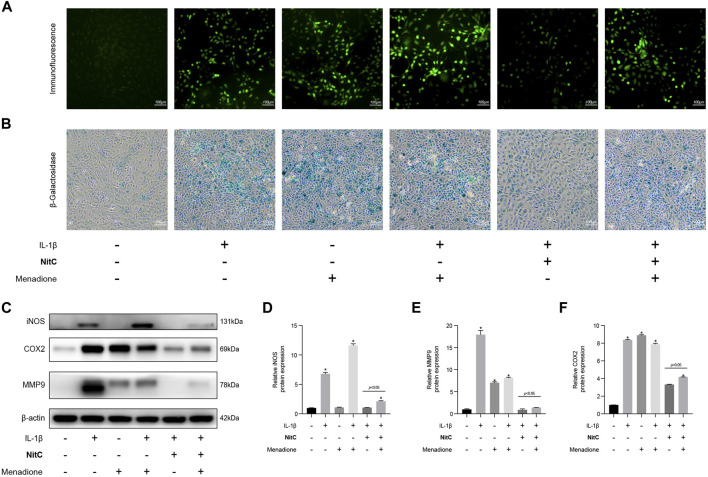
Regeneration of ROS rescue the impact of NitC on RACs. **(A)** The ROS levels in RACs were detected through immunofluorescence analysis. **(B)** β-Galactosidase activity assay was performed to identify the senescent RACs. **(C)** WB analysis of iNOS, COX2, MMP9, and β-Actin. The relative protein expression was quantified and shown respectively in **(D–F)**. ∗*p* < 0.05 versus the model group, other significant differences between the groups were shown as indicated in the figure.

To investigate the underlying mechanism of the NitC effect on IL-1β-Induced RACs, menadione, a synthetic naphthoquinone widely used as a free radical generator, was exerted to further experiments. For menadione stimulation, RACs were cultured on 6-well plates and pretreated with 50 μM menadione for 1 h according to recent studies (Wang et al., 2020). Firstly, immunofluorescence analysis was performed to detect the ROS levels in RACs. The results probed that intervention of menadione significantly increased the ROS generation and managed to regenerate ROS in NitC-pretreated RACs (Figure 5A). Furthermore, the β-Galactosidase activity assay results showed that both IL-1β and menadione stimulation-induced cellular senescence in RACs, whereas menadione treatment was able to restore the effect of NitC on IL-1β-Induced RACs (Figure 5B), indicating NitC ameliorated senescence in RACs *via* scavenging ROS. Interestingly, Western blot analysis revealed that regeneration of ROS by menadione could significantly rescue the anti-inflammatory effect of NitC on IL-1β-Induced RACs through upregulation of iNOS, COX2 and MMP9 (Figures 5C–F). Altogether these results identified the potential underlying mechanism of NitC was scavenging ROS in RACs under pathological conditions.

### NitC Suppressed NLRP3 Inflammasomes Activation in RAW 264.7 Through Scavenging ROS

**FIGURE 6:**
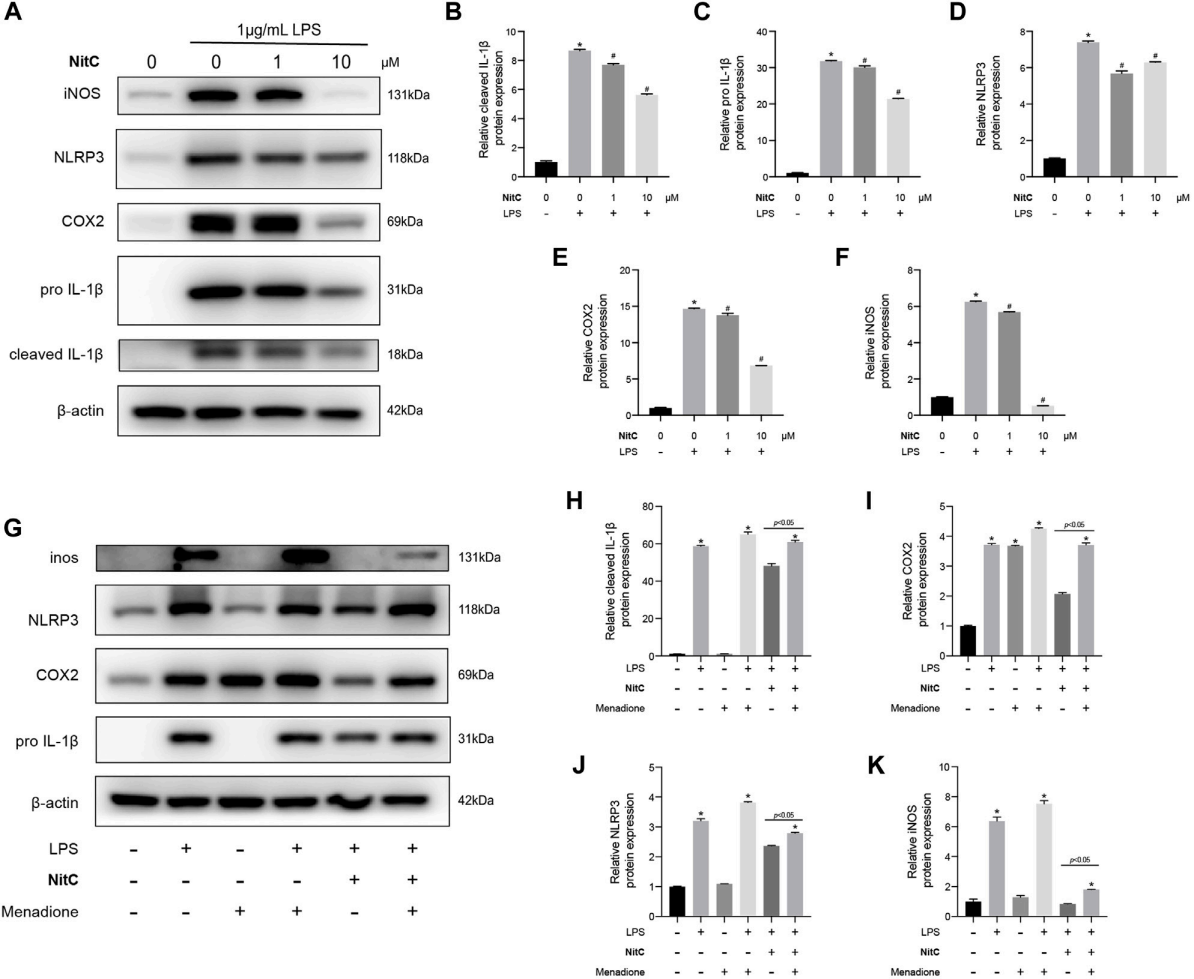
NitC inhibited NLRP3-inflammasome activation through scavenging ROS. **(A)** The WB analysis and quantitative analysis of the **(B)** cleaved IL-1β, **(C)** pro-IL-1β, **(D)** NLRP3, **(E)** COX2, and **(F)** iNOS. **(G)** Another WB analysis was performed to investigate the role of ROS in NitC-treated RAW 264.7. The quantified results of **(H)** pro-IL-1β, **(I)** COX2, **(J)** NLRP3, and **(K)** iNOS were shown in the figure. ∗*p* < 0.05 versus the model group, other significant differences between the groups were shown as indicated in the figure.

With the advance in the understanding of OA pathophysiology, OA has now been considered a disease of the whole joint (Martel-Pelletier et al., 2016). Among the structural alterations in the joint, synovial membrane inflammation is another independent driver of OA onset (Mathiessen and Conaghan, 2017). Jin et al. (2011) reported that besides secreting catabolic cytokine IL-1β, NLRP3 inflammasome activations mediated ectopic deposition of hydroxyapatite crystals in joints, playing an essential role in the pathogenesis of OA. In the present study, we further investigated the role of NitC on LPS-induced RAW 264.7, a well-established macrophage cell line wildly used in OA investigations (Lv et al., 2021). The Western blot analysis showed that LPS stimulation for 24 h remarkably upregulated the protein expression of iNOS, COX2, IL-1β, and NLRP3, indicating the activations of the NLRP3 inflammasome, whereas NitC treatment significantly inhibited the effect in a dose-dependent manner (Figures 6A–F). Previous studies demonstrated the crucial role of the ROS-NLRP3-Inflammasome axis in other disease progressions, nevertheless, it has not been reported in OA yet (Xie et al., 2020). Here, we found regeneration of ROS by menadione could significantly reduce the effect of NitC on LPS-induced NLRP3 inflammasome activations (Figures 6G–K). In conclusion, our results demonstrated NitC was capable of suppressing NLRP3 inflammasome activation in LPS-induced RAW 264.7 through scavenging ROS.

### NitC Alleviated OA Progression in DMM Rats

**FIGURE 7:**
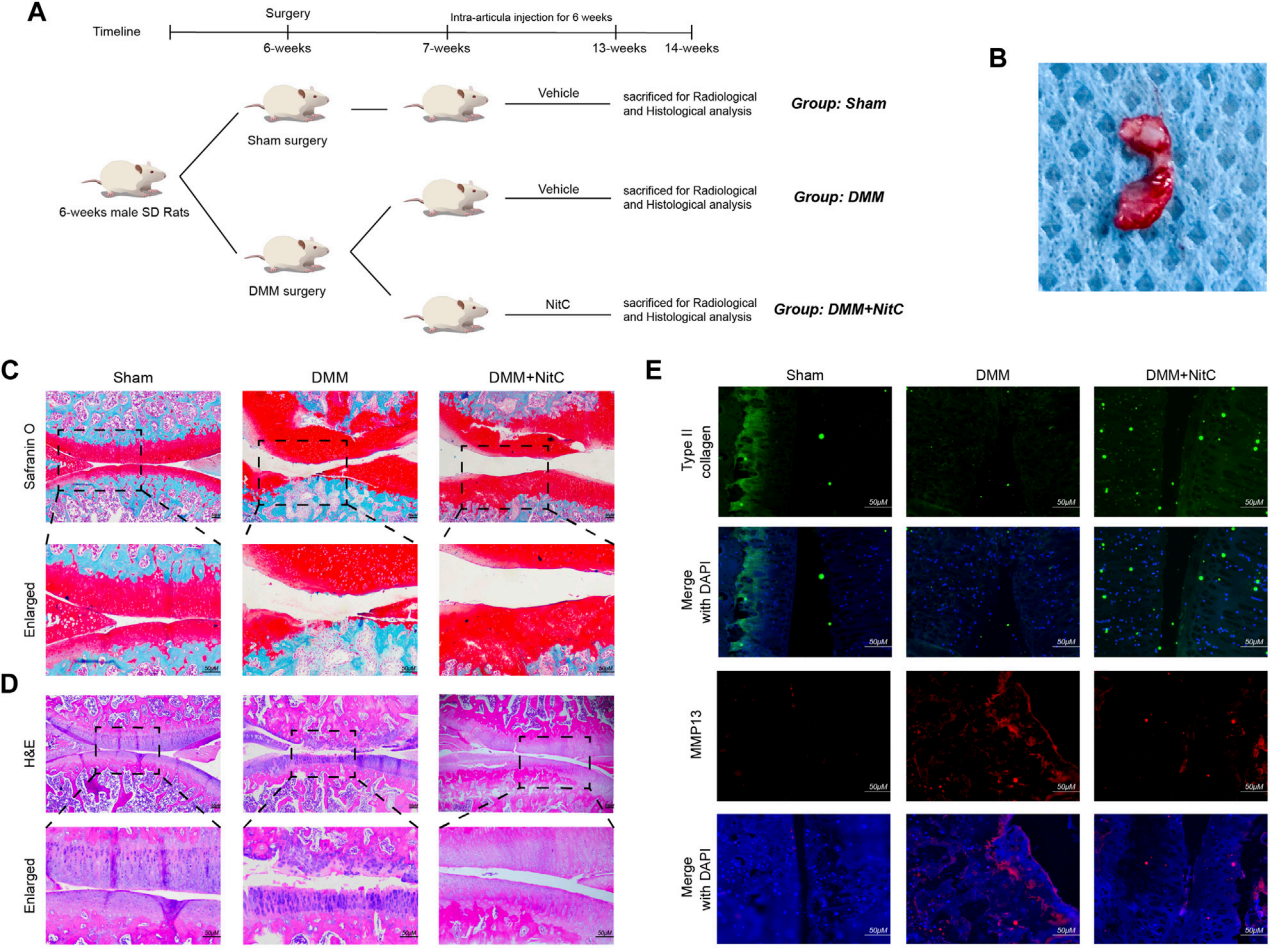
Intra-articular injection of NitC ameliorated OA progression in rat OA model. **(A)** The flowchart for animal procedures. **(B)** The resected medial meniscus. **(C)** Microscopic images of safranin O staining of rat knee joints. **(D)** Representative images of HE staining of rat knee joints. **(E)** Typical images of immunofluorescence analysis against type II collagen and MMP13. Scale bars = 200 μM.

As for *in vivo* assessment of cartilage degeneration, rat OA models were induced by surgical resection of the medial meniscus. The flowchart for animal procedures was shown as in Figure 7A. Briefly, twenty-four SD rats were randomly divided into three groups. Rats in the Sham group underwent sham surgery while other rats underwent DMM surgery to resect the medial meniscus (Figure 7B). The safranin O staining results showed notable loss of superficial articular cartilage in the DMM group, while intra-articular injection of NitC rescued it (Figure 7C). Aligned with the safranin O staining, the HE staining results showed remarkable cartilage degradation in the DMM group, whereas NitC treatment alleviated the degradation (Figure 7D). Additionally, the results of immunofluorescence analysis showed that type II collagen expression was highly decreased while MMP13 expression was increased in the DMM group, while NitC treatment reversed the effect (Figure 7E). Collectively, these results demonstrated that intra-articular injection of NitC was capable of ameliorating OA progression in the rat OA model.

### NitC Ameliorated the Radiological Features of OA

**FIGURE 8:**
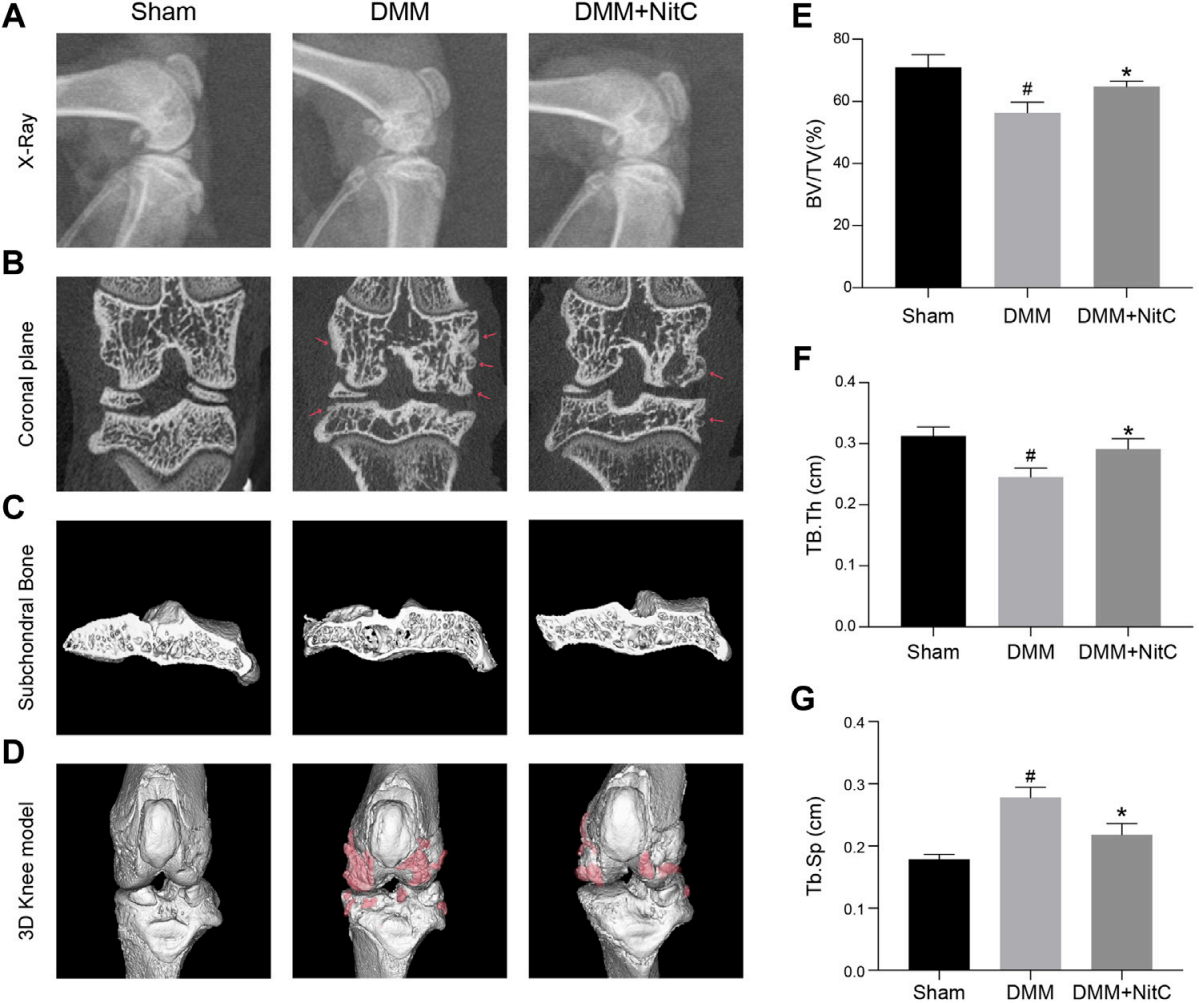
Radiological assessment of NitC impact on DMM rats. **(A)** Representative images of rat knees X-ray scanning. The treatment was performed as previously described in **Methods and Materials**. **(B)** The coronal plane of rat knees. The red arrow showed osteophyte formation. **(C)** Subchondral bone in each group. **(D)** Reconstruction of 3D rat knee models. IMALYTICS Preclinical 2.1 software was used to evaluate BV/TV **(E)**, Tb.Th **(F)**, and Tb.Sp **(G)**. *#p* < 0.05 versus the control group. ∗*p* < 0.05 versus the model group.

We further performed X-ray and micro-CT analysis to exert radiological assessment of alterations in rat joints. X-ray results revealed characteristic narrowing joint space and irregular contours of the articular surface in DMM models, while intra-articular injection of NitC significantly ameliorated these osteoarthritis progression features (Figure 8A). Moreover, the coronal plane of micro-CT results indicated that rats in DMM groups developed marginal osteophytes around the articular surface, while the treatment of NitC reduced the osteophytes (Figure 8B; Red arrow noted the typical osteophytes). 3D reconstruction of tibial subchondral bone structures showed the formation of bone cysts, one of the common features of advanced OA, in the DMM models group, whereas the intervention of NitC restored it (Figure 8C). The whole joint 3D reconstruction was established to observe marginal osteophytes and other alterations, demonstrating NitC treatment could remarkably restore the OA progression (Figure 8D; red parts noted the OA alterations). Furthermore, three trabecular bone indices including trabecular bone volume fraction (BV/TV; %), trabecular thickness (Tb.Th; cm) and trabecular spacing (Tb.Sp; cm) were calculated in the present study. The results showed DMM surgery significantly decreased BV/TV (Figure 8E) and Tb.Th (Figure 8F), meanwhile increased Tb.Sp (Figure 8G), while NitC treatment alleviated the alterations, indicating NitC treatment might preserve the tibial subchondral bone microarchitecture in OA progression. Taken together, our results demonstrated that NitC treatment remarkably ameliorated the radiological features in advanced OA.

## Discussion

Given the rapid progress of the aging and obese population globally, the OA prevalence is unprecedently rising than in previous decades (Hunter and Bierma-Zeinstra, 2019). To date, pharmaceutical treatments recommended and used in clinical works are merely symptomatically, namely no drugs are capable of modulating OA progression and eventually reducing long-term disability, representing a major health challenge for the coming future (Jones et al., 2019; Latourte et al., 2020). Recently, collecting insights into the pathogenesis of OA has revealed that alterations in cartilage, subchondral bone, synovial membrane, meniscus, ligament and other tissues accumulate gradually, ultimately leading to joint dysfunction (Martel-Pelletier et al., 2016).

Further unraveling of the underlying mechanism showed OA was a multifactorial disorder, in which chronic inflammation in tissues plays a vital role (Liu-Bryan and Terkeltaub, 2015; Robinson et al., 2016). Hence, we firstly examined the effect of NitC on chondrocytes inflammation. iNOS and COX-2, known as the positive regulator of inflammation (Matziouridou et al., 2018; Chen et al., 2021), were remarkably downregulated following NitC treatment in IL-1β-Induced OA models *in vitro*. Furthermore, the intervention of NitC suppressed the activation of MAPK and NF-κB cell signaling pathways in the OA progression. Enhancing catabolic metabolism in ECM, including upregulation of MMPs and degradation of collagens, is a pivotal factor for OA initiation and development (Rahmati et al., 2017). Western blot, qRT-PCR, Safranin-O staining, Alcian Blue staining, immunofluorescence and other diverse experiments analysis in the present study congruently indicated that NitC treatment was capable of inhibiting MMPs expression, reversing collagen degradation and eventually rescuing cartilage erosion.

To further explore the underlying mechanism, we utilized a series of emerging bioinformatics tools: PharmMapper, STRING and Cytoscape [25–30, 47] The predictive results showed the top 10 proteins that probably directly bind with NitC. Interestingly, among these 10 proteins, eight proteins were reported to be involved with reactive oxygen species (ROS) generation or clearance (Blaser et al., 2016; Matziouridou et al., 2018; Gilardini Montani et al., 2019; Bermúdez-Muñoz et al., 2020; Chen et al., 2021). Meanwhile, previous studies demonstrated that NitC could inhibit cytochrome P450 family enzymes (Mao et al., 2018; Mao et al., 2019), which play a central role in ROS generation (Leung and Nieto, 2013). All of these results indicated NitC possesses the potential capacity of modulating ROS. Our flow cytometry and immunofluorescence result further probed that NitC treatment inhibited IL-1β-Induced ROS generation in RACs.

The ROS production in osteoarthritis tissues has received increasing attention. Excess levels of ROS lead to cartilage degradation, oxidative damage as well as redox-regulated cell signaling pathway disruption (Bai et al., 2019; Bolduc et al., 2019). Additionally, in its role as an oxidative stress stimulation, overloaded ROS induces chondrocytes cellular senescence and macrophage activation (Jin et al., 2011; Pei et al., 2019; Au et al., 2020). Intervention with Menadione, a synthetic naphthoquinone widely used as a free radical generator, was exerted to further confirm the crucial role of ROS in determining chondrocyte and macrophage destiny (Wang et al., 2020). Consistently, our results demonstrated that NitC was able to alleviate chondrocyte cellular senescence *via* scavenging ROS. Meanwhile, regeneration of ROS reduce NitC-treated downregulation of MMP9 and proinflammatory mediators including iNOS and COX2, manifesting the inter-dependent relationship between oxidative stress and inflammation in OA progression (Ansari et al., 2020).

Asides from effects on chondrocytes, ROS production appears a dispensable role for NLRP3 inflammasome activation according to recent studies (He et al., 2016; Chen et al., 2019). NLRP3 inflammasome has been implicated in osteoarthritis pathological progression through secreting proinflammatory cytokines including IL-1β and tumor necrosis factor-alpha (TNF-a), producing cartilage degrading enzymes like MMP3 and aggravating synovial membrane inflammation (Mathiessen and Conaghan, 2017; McAllister et al., 2018; Chen et al., 2019). In this study, we found that NitC remarkably suppressed the expression of LPS-stimulated inflammation-related genes and inactivated NLRP3 inflammasome in RAW264.7 *via* clearance of ROS, manifesting another protective aspect in alleviating OA progression.

The advantages of radiological assessments of rat knee joints were fully taken in the present study. X-ray results revealed narrowing joint space and irregular contours of the articular surface in the DMM model, while intra-articular injection of NitC significantly ameliorated these osteoarthritis progression features. More precise observations by micro-CT showed fewer marginal osteophytes around the articular surface in the NitC-treated group. Improvements in the joint osteoarthritis alterations were shown by representative images of knee joint 3D reconstruction. Analyzing data from subchondral bone demonstrated that NitC intervention significantly reversed osteoarthritic changes of tibial subchondral trabecular bone. All of these results supported that NitC treatment might preserve the tibial subchondral bone microarchitecture in OA progression. Aligned with radiological measurement, other *in vivo* experiments showed NitC was capable of maintaining cartilage structural integrity, suppressing inflammation and inhibiting ECM degradation.

The present study reported the protective role of NitC in murine osteoarthritis models both *in vitro* and *in vivo* for the first time. In terms of the complicated pathogenesis of OA, we not only investigated chondrocytes but also demonstrated the effect of NitC on macrophages. Besides, several acknowledged vital phenotypes of OA, including inflammatory mediator secretion, ECM degradation, chondrocytes cellular senescence, oxidative stress, NLRP3 inflammasome activation, and subchondral bone alterations were comprehensively and substantially investigated in this study. Another highlight of this study is providing a plausible example for understanding and utilizing the emerging bioinformatics tools with the rapid advance in computer technology. Frankly, a few limitations of this study should be noted. Firstly, our findings merely indicated that inactivation of the MAPK/NF-κB pathway was involved in the effect of NitC on inducing osteoarthritic chondrocytes. Whether MAPK and NF-κB pathways are the direct targets of NitC remains elusive. Secondly, although RAW 264.7 cell line has been widely used in studying osteoarthritis as the synovial membrane macrophage, it is still not the exact cell type residing in the joint tissues. Furthermore, more works should be focused on the cross-talk between chondrocytes and macrophages which was lacking in the study. Lastly, our finding demonstrated that NitC was capable of scavenging ROS in both chondrocytes and macrophages. Further investigations should be conducted to illustrate whether the specific underlying mechanism was similar to the previous studies through inhibiting cytochrome P450 family enzymes or through other distinct regulatory mechanisms.

## Conclusion

In the present study, we reported first that NitC was capable of inhibiting enhanced catabolic response in the extracellular matrix, downregulating secretion of inflammatory mediators and inactivating of MAPK/NF-kB pathway in OA progression. In addition to the anti-inflammatory effect, NitC exerted its anti-oxidative activity through ameliorating cellular senescence, reducing oxidative stress and inhibiting NLRP3 inflammasome activation *via* scavenging reactive oxygen species (ROS). Based on these *in vitro* and *in vivo* results, we demonstrated the promising role of NitC as a drug treatment for OA.

## Data Availability

The original contributions presented in the study are included in the article/Supplementary Material, further inquiries can be directed to the corresponding authors.

## References

[B1] Ansari M. Y. Ahmad N. Haqqi T. M. (2020). Oxidative Stress and Inflammation in Osteoarthritis Pathogenesis: Role of Polyphenols. Biomed. Pharmacother. 129, 110452. 10.1016/j.biopha.2020.110452 32768946 PMC8404686

[B2] Au M. Liu Z. Rong L. Zheng Y. Wen C. (2020). Endothelin-1 Induces Chondrocyte Senescence and Cartilage Damage via Endothelin Receptor Type B in a Post-Traumatic Osteoarthritis Mouse Model. Osteoarthr. Cartil. 28, 1559–1571. 10.1016/j.joca.2020.08.006 32858189

[B3] Bai Y. Gong X. Dou C. Cao Z. Dong S. (2019). Redox Control of Chondrocyte Differentiation and Chondrogenesis. Free Radic. Biol. Med. 132, 83–89. 10.1016/j.freeradbiomed.2018.10.443 30394290

[B4] Bao J. Lin C. Zhou X. Ma D. Ge L. Xu K. (2021). CircFAM160A2 Promotes Mitochondrial Stabilization and Apoptosis Reduction in Osteoarthritis Chondrocytes by Targeting miR-505-3p and SIRT3. Oxid. Med. Cell. Longev. 2021, 5712280. 10.1155/2021/5712280 34646424 PMC8505077

[B5] Bermúdez-Muñoz J. M. Celaya A. M. Hijazo-Pechero S. Wang J. Serrano M. Varela-Nieto I. (2020). G6PD Overexpression Protects from Oxidative Stress and Age-Related Hearing Loss. Aging Cell 19, e13275–18. 10.1111/acel.13275 33222382 PMC7744953

[B6] Blaser H. Dostert C. Mak T. W. Brenner D. (2016). TNF and ROS Crosstalk in Inflammation. Trends Cell Biol. 26, 249–261. 10.1016/j.tcb.2015.12.002 26791157

[B7] Bolduc J. A. Collins J. A. Loeser R. F. (2019). Reactive Oxygen Species, Aging and Articular Cartilage Homeostasis. Free Radic. Biol. Med. 132, 73–82. 10.1016/j.freeradbiomed.2018.08.038 30176344 PMC6342625

[B8] Chen Z. Zhong H. Wei J. Lin S. Zong Z. Gong F. (2019). Inhibition of Nrf2/HO-1 Signaling Leads to Increased Activation of the NLRP3 Inflammasome in Osteoarthritis. Arthritis Res. Ther. 21, 300–313. 10.1186/s13075-019-2085-6 31870428 PMC6929452

[B9] Chen H. Qian Z. Zhang S. Tang J. Fang L. Jiang F. (2021). Silencing COX-2 Blocks PDK1/TRAF4-Induced AKT Activation to Inhibit Fibrogenesis During Skeletal Muscle Atrophy. Redox Biol. 38, 101774. 10.1016/j.redox.2020.101774 33152664 PMC7645269

[B10] Conaghan P. G. Cook A. D. Hamilton J. A. Tak P. P. (2019). Therapeutic Options for Targeting Inflammatory Osteoarthritis Pain. Nat. Rev. Rheumatol. 15, 355–363. 10.1038/s41584-019-0221-y 31068673

[B11] Coryell P. R. Diekman B. O. Loeser R. F. (2021). Mechanisms and Therapeutic Implications of Cellular Senescence in Osteoarthritis. Nat. Rev. Rheumatol. 17, 47–57. 10.1038/s41584-020-00533-7 33208917 PMC8035495

[B12] Cui Y. Wu L. Cao R. Xu H. Xia J. Wang Z. P. (2020). Antitumor Functions and Mechanisms of Nitidine Chloride in Human Cancers. J. Cancer 11, 1250–1256. 10.7150/jca.37890 31956371 PMC6959075

[B13] Gilardini Montani M. S. Santarelli R. Granato M. Gonnella R. Torrisi M. R. Faggioni A. (2019). EBV Reduces Autophagy, Intracellular ROS and Mitochondria to Impair Monocyte Survival and Differentiation. Autophagy 15, 652–667. 10.1080/15548627.2018.1536530 30324853 PMC6526866

[B14] Guo Y. N. Cui S. J. Tian Y. J. Zhao N. R. Zhang Y. D. Gan Y. H. (2022). Chondrocyte Apoptosis in Temporomandibular Joint Osteoarthritis Promotes Bone Resorption by Enhancing Chemotaxis of Osteoclast Precursors. Osteoarthr. Cartil. 10.1016/j.joca.2022.04.002 35513247

[B15] He Y. Hara H. Núñez G. (2016). Mechanism and Regulation of NLRP3 Inflammasome Activation. Trends biochem. Sci. 41, 1012–1021. 10.1016/j.tibs.2016.09.002 27669650 PMC5123939

[B16] Hunter D. J. Bierma-Zeinstra S. (2019). Osteoarthritis. Lancet 393, 1745–1759. 10.1016/S0140-6736(19)30417-9 31034380

[B17] Jeon O. H. Kim C. Laberge R. M. Demaria M. Rathod S. Vasserot A. P. (2017). Local Clearance of Senescent Cells Attenuates the Development of Post-Traumatic Osteoarthritis and Creates a Pro-regenerative Environment. Nat. Med. 23, 775–781. 10.1038/nm.4324 28436958 PMC5785239

[B18] Jin C. Frayssinet P. Pelker R. Cwirka D. Hu B. Vignery A. (2011). NLRP3 Inflammasome Plays a Critical Role in the Pathogenesis of Hydroxyapatite-Associated Arthropathy. Proc. Natl. Acad. Sci. U. S. A. 108, 14867–14872. 10.1073/pnas.1111101108 21856950 PMC3169126

[B19] Jones I. A. Togashi R. Wilson M. L. Heckmann N. Vangsness C. T. (2019). Intra-Articular Treatment Options for Knee Osteoarthritis. Nat. Rev. Rheumatol. 15, 77–90. 10.1038/s41584-018-0123-4 30498258 PMC6390843

[B20] Kang D. Lee J. Jung J. Carlson B. A. Chang M. J. Chang C. B. (2022). Selenophosphate Synthetase 1 Deficiency Exacerbates Osteoarthritis by Dysregulating Redox Homeostasis. Nat. Commun. 13, 1–14. 10.1038/s41467-022-28385-7 35140209 PMC8828855

[B21] Katz J. N. Arant K. R. Loeser R. F. (2021). Diagnosis and Treatment of Hip and Knee Osteoarthritis: A Review. JAMA - J. Am. Med. Assoc. 325, 568–578. 10.1001/jama.2020.22171 PMC8225295 33560326

[B22] Krishnan Y. Grodzinsky A. J. (2018). Cartilage Diseases. Matrix Biol. 71-72, 51–69. 10.1016/j.matbio.2018.05.005 29803938 PMC6146013

[B23] Latourte A. Kloppenburg M. Richette P. (2020). Emerging Pharmaceutical Therapies for Osteoarthritis. Nat. Rev. Rheumatol. 16, 673–688. 10.1038/s41584-020-00518-6 33122845

[B24] Leung T. M. Nieto N. (2013). CYP2E1 and Oxidant Stress in Alcoholic and Non-Alcoholic Fatty Liver Disease. J. Hepatol. 58, 395–398. 10.1016/j.jhep.2012.08.018 22940046

[B25] Li L. Tu M. Yang X. Sun S. Wu X. Zhou H. (2014). The Contribution of Human OCT1, OCT3, and CYP3A4 to Nitidine Chloride-Induced Hepatocellular Toxicity. Drug Metab. Dispos. 42, 1227–1234. 10.1124/dmd.113.056689 24778366

[B26] Liang R. Zhao J. Li B. Cai P. Loh X. J. Xu C. (2020). Implantable and Degradable Antioxidant Poly(ε-Caprolactone)-Lignin Nanofiber Membrane for Effective Osteoarthritis Treatment. Biomaterials 230, 119601. 10.1016/j.biomaterials.2019.119601 31711715

[B27] Liu X. Ouyang S. Yu B. Liu Y. Huang K. Gong J. (2010). PharmMapper Server: A Web Server for Potential Drug Target Identification Using Pharmacophore Mapping Approach. Nucleic Acids Res. 38, W609–W614. 10.1093/nar/gkq300 20430828 PMC2896160

[B28] Liu-Bryan R. Terkeltaub R. (2015). Emerging Regulators of the Inflammatory Process in Osteoarthritis. Nat. Rev. Rheumatol. 11, 35–44. 10.1038/nrrheum.2014.162 25266449 PMC4374654

[B29] Lv Z. Xu X. Sun Z. Yang Y. X. Guo H. Li J. (2021). TRPV1 Alleviates Osteoarthritis by Inhibiting M1 Macrophage Polarization via Ca2+/CaMKII/Nrf2 Signaling Pathway. Cell Death Dis. 12, 504. 10.1038/s41419-021-03792-8 34006826 PMC8131608

[B30] Ma C. Zhou X. Xu K. Wang L. Yang Y. Wang W. (2018). Specnuezhenide Decreases Interleukin-1β-Induced Inflammation in Rat Chondrocytes and Reduces Joint Destruction in Osteoarthritic Rats. Front. Pharmacol. 9, 700–712. 10.3389/fphar.2018.00700 30050432 PMC6052343

[B31] Mao X. Hu Z. Wang Q. Zhang N. Zhou S. Peng Y. (2018). Nitidine Chloride Is a Mechanism-Based Inactivator of CYP2D6. Drug Metab. Dispos. 46, 1137–1145. 10.1124/dmd.117.079780 29773554

[B32] Mao X. Wang J. Wang Q. Yang L. Li Y. Lin H. (2019). Nitidine Chloride-Induced CYP1 Enzyme Inhibition and Alteration of Estradiol Metabolism. Drug Metab. Dispos. 47, 919–927. 10.1124/dmd.119.086892 31147316

[B33] Martel-Pelletier J. Barr A. J. Cicuttini F. M. Conaghan P. G. Cooper C. Goldring M. B. (2016). Osteoarthritis. Nat. Rev. Dis. Prim. 2, 16072. 10.1038/nrdp.2016.72 27734845

[B34] Mathiessen A. Conaghan P. G. (2017). Synovitis in Osteoarthritis: Current Understanding with Therapeutic Implications. Arthritis Res. Ther. 19, 18–19. 10.1186/s13075-017-1229-9 28148295 PMC5289060

[B35] Matziouridou C. Rocha S. D. C. Haabeth O. A. Rudi K. Carlsen H. Kielland A. (2018). INOS- and NOX1-Dependent ROS Production Maintains Bacterial Homeostasis in the Ileum of Mice. Mucosal Immunol. 11, 774–784. 10.1038/mi.2017.106 29210363

[B36] McAllister M. J. Chemaly M. Eakin A. J. Gibson D. S. McGilligan V. E. (2018). NLRP3 as a Potentially Novel Biomarker for the Management of Osteoarthritis. Osteoarthr. Cartil. 26, 612–619. 10.1016/j.joca.2018.02.901 29499288

[B37] Mehana E. E. Khafaga A. F. El-Blehi S. S. (2019). The Role of Matrix Metalloproteinases in Osteoarthritis Pathogenesis: An Updated Review. Life Sci. 234, 116786. 10.1016/j.lfs.2019.116786 31445934

[B38] Mobasheri A. Rayman M. P. Gualillo O. Sellam J. Van Der Kraan P. Fearon U. (2017). The Role of Metabolism in the Pathogenesis of Osteoarthritis. Nat. Rev. Rheumatol. 13, 302–311. 10.1038/nrrheum.2017.50 28381830

[B39] Moqbel S. A. A. He Y. Xu L. Ma C. Ran J. Xu K. (2020). Rat Chondrocyte Inflammation and Osteoarthritis Are Ameliorated by Madecassoside. Oxid. Med. Cell. Longev. 2020, 7540197. 10.1155/2020/7540197 32089778 PMC7023724

[B40] O’ Neill T. W. McCabe P. S. McBeth J. (2018). Update on the Epidemiology, Risk Factors and Disease Outcomes of Osteoarthritis. Best Pract. Res. Clin. Rheumatology 32, 312–326. 10.1016/j.berh.2018.10.007 30527434

[B41] Otasek D. Morris J. H. Bouças J. Pico A. R. Demchak B. (2019). Cytoscape Automation: Empowering Workflow-Based Network Analysis. Genome Biol. 20, 185–215. 10.1186/s13059-019-1758-4 31477170 PMC6717989

[B42] Park S. Arai Y. Bello A. Park H. Kim D. Park K. S. (2021). SPRY4 Acts as an Indicator of Osteoarthritis Severity and Regulates Chondrocyte Hypertrophy and ECM Protease Expression. Npj Regen. Med. 6, 56–59. 10.1038/s41536-021-00165-9 34535669 PMC8448831

[B43] Pei Y. Cui F. Du X. Shang G. Xiao W. Yang X. (2019). Antioxidative Nanofullerol Inhibits Macrophage Activation and Development of Osteoarthritis in Rats. Int. J. Nanomed. 14, 4145–4155. 10.2147/IJN.S202466 PMC6559768 31239673

[B44] Rahmati M. Nalesso G. Mobasheri A. Mozafari M. (2017). Aging and Osteoarthritis: Central Role of the Extracellular Matrix. Ageing Res. Rev. 40, 20–30. 10.1016/j.arr.2017.07.004 28774716

[B45] Ran J. Ma C. Xu K. Xu L. He Y. Moqbel S. A. A. (2018). Schisandrin B Ameliorated Chondrocytes Inflammation and Osteoarthritis via Suppression of NF-κB and MAPK Signal Pathways. Drug Des. Devel. Ther. 12, 1195–1204. 10.2147/DDDT.S162014 PMC5953308 29785089

[B46] Robinson W. H. Lepus C. M. Wang Q. Raghu H. Mao R. Lindstrom T. M. (2016). Low-Grade Inflammation as a Key Mediator of the Pathogenesis of Osteoarthritis. Nat. Rev. Rheumatol. 12, 580–592. 10.1038/nrrheum.2016.136 27539668 PMC5500215

[B47] Shannon P. Markiel A. Ozier O. Baliga N. S. Wang J. T. Ramage D. (2003). Cytoscape: A Software Environment for Integrated Models of Biomolecular Interaction Networks. Genome Res. 13, 2498–2504. 10.1101/gr.1239303 14597658 PMC403769

[B48] Szklarczyk D. Gable A. L. Lyon D. Junge A. Wyder S. Huerta-Cepas J. (2019). STRING V11: Protein-Protein Association Networks with Increased Coverage, Supporting Functional Discovery in Genome-Wide Experimental Datasets. Nucleic Acids Res. 47, D607–D613. 10.1093/nar/gky1131 30476243 PMC6323986

[B49] Tudorachi N. B. Totu E. E. Fifere A. Ardeleanu V. Mocanu V. Mircea C. (2021). The Implication of Reactive Oxygen Species and Antioxidants in Knee Osteoarthritis. Antioxidants (Basel) 10, 1–29. 10.3390/antiox10060985 PMC8233827 34205576

[B50] Wang Z. Jiang W. Zhang Z. Qian M. Du B. (2012). Nitidine Chloride Inhibits LPS-Induced Inflammatory Cytokines Production via MAPK and NF-kappaB Pathway in RAW 264.7 Cells. J. Ethnopharmacol. 144, 145–150. 10.1016/j.jep.2012.08.041 22971898

[B51] Wang X. Pan C. Gong J. Liu X. Li H. (2016). Enhancing the Enrichment of Pharmacophore-Based Target Prediction for the Polypharmacological Profiles of Drugs. J. Chem. Inf. Model. 56, 1175–1183. 10.1021/acs.jcim.5b00690 27187084

[B52] Wang X. Shen Y. Wang S. Li S. Zhang W. Liu X. (2017). PharmMapper 2017 Update: A Web Server for Potential Drug Target Identification with a Comprehensive Target Pharmacophore Database. Nucleic Acids Res. 45, W356–W360. 10.1093/nar/gkx374 28472422 PMC5793840

[B53] Wang Y. Zhao X. Liu-Bryan R. (2020). Role of TLR2 and TLR4 in Regulation of Articular Chondrocyte Homeostasis. Osteoarthr. Cartil. 28, 669–674. 10.1016/j.joca.2020.01.011 PMC7214200 32007503

[B54] Xie C. Ge M. Jin J. Xu H. Mao L. Geng S. (2020). Mechanism Investigation on Bisphenol S-Induced Oxidative Stress and Inflammation in Murine RAW264.7 Cells: The Role of NLRP3 Inflammasome, TLR4, Nrf2 and MAPK. J. Hazard. Mater. 394, 122549. 10.1016/j.jhazmat.2020.122549 32283380

[B55] Xu L. Wu Z. He Y. Chen Z. Xu K. Yu W. (2020). MFN2 Contributes to Metabolic Disorders and Inflammation in the Aging of Rat Chondrocytes and Osteoarthritis. Osteoarthr. Cartil. 28, 1079–1091. 10.1016/j.joca.2019.11.011 32416221

[B56] Xu K. Lin C. Ma D. Chen M. Zhou X. He Y. (2021). Spironolactone Ameliorates Senescence and Calcification by Modulating Autophagy in Rat Tendon-Derived Stem Cells via the NF-κB/MAPK Pathway. Oxid. Med. Cell. Longev. 2021, 1–15. 10.1155/2021/5519587 PMC8263237 34306308

[B57] Yang N. Yue R. Ma J. Li W. Zhao Z. Li H. (2019). Nitidine Chloride Exerts Anti-Inflammatory Action by Targeting Topoisomerase I and Enhancing IL-10 Production. Pharmacol. Res. 148, 104368. 10.1016/j.phrs.2019.104368 31415918

[B58] Yeo C. Ahn C. R. Kim J. E. Kim Y. W. Park J. Ahn K. S. (2022). Chaenomeles Fructus (CF), the Fruit of Chaenomeles Sinensis Alleviates IL-1β Induced Cartilage Degradation in Rat Articular Chondrocytes. Int. J. Mol. Sci. 23, 4360. 10.3390/ijms23084360 35457176 PMC9025567

