# Supplementary material for: Nitidine Chloride Alleviates Inflammation and Cellular Senescence in Murine Osteoarthritis Through Scavenging ROS
Source: Front Pharmacol. 2022 Jul 22;13:919940. doi: 10.3389/fphar.2022.919940 (PMC9353946; doi:10.3389/fphar.2022.919940)
Supplement: Supplementary file 1 [file DataSheet1.docx]

| Supplementary Table1: primers for qRT-PCR | | |
| --- | --- | --- |
| Gene | Forward | Reverse |
| 18S | CCTGAGAAACGGCTACCACA | ACCAGACTTGCCCTCCAATG |
| COL2 | GCCAGGATGCCCGAAAATTAG | GTCACCTCTGGGTCCTTGTTC |
| COX2 | GTGGAAAAGCCTCGTCCAGA | TCCTCCGAAGGTGCTAGGTT |
| iNOS | AGTCAACTACAAGCCCCACG | AGAAACTTCCAGGGGCAAGC |
| MMP9 | GATCCCCAGAGCGTTACTCG | GTTGTGGAAACTCACACGCC |
| MMP13 | CTGGGCCCTGAATGGGTATG | CTCAAAGTGAACCGCAGCAC |

| SupplementaryTable2: predictive results from PharmMapper | | | | |
| --- | --- | --- | --- | --- |
| Pharma Model | Fit | Norm Fit | Name | Uniplot |
| 2lqi_B_cavity_1 | 3.37 | 0.8426 | NONE | NONE |
| 1rf8_A_cavity_2 | 3.201 | 0.8003 | Eukaryotic translation initiation factor 4E | P07260 |
| 2co9_A_cavity_1 | 3.169 | 0.7923 | Thymocyte selection-associated high mobility group box protein TOX | Q66JW3 |
| 2hz7_A_cavity_3 | 3.076 | 0.769 | Glutaminyl-tRNA synthetase | P56926 |
| 2c0p_A_cavity_2 | 3.81 | 0.7619 | Acetylcholinesterase | P21836 |
| 1win_A_cavity_1 | 3.752 | 0.7503 | Flotillin-2 | Q60634 |
| 3fqd_A_cavity_8 | 3.715 | 0.743 | 5-3 exoribonuclease 2 | P40848 |
| 2aeu_A_cavity_4 | 2.967 | 0.7418 | UPF0425 pyridoxal phosphate-dependent protein MJ0158 | Q57622 |
| 1lsh_B_cavity_3 | 2.953 | 0.7383 | Vitellogenin | Q91062 |
| 1x65_A_cavity_1 | 4.424 | 0.7373 | Cold shock domain-containing protein E1 | O75534 |
| 1ger_A_cavity_3 | 2.871 | 0.7178 | Glutathione reductase | P06715 |
| 1w07_B_cavity_5 | 2.832 | 0.708 | Acyl-coenzyme A oxidase 1, peroxisomal | O65202 |
| 2cop_A_cavity_3 | 2.818 | 0.7044 | Acyl-CoA-binding domain-containing protein 6 | Q9BR61 |
| 2ron_A_cavity_1 | 2.778 | 0.6946 | Surfactin synthetase thioesterase subunit | Q08788 |
| 1fgu_B_cavity_1 | 2.759 | 0.6898 | Replication protein A 70 kDa DNA-binding subunit | P27694 |
| 1wde_A_cavity_2 | 3.41 | 0.6819 | Probable diphthine synthase | Q9YDI2 |
| 2q2e_A_cavity_2 | 2.674 | 0.6686 | Type II DNA topoisomerase VI subunit A | Q8PUB7 |
| 1wym_A_cavity_1 | 2.627 | 0.6568 | Transgelin-2 | P37802 |
| 1yav_B_cavity_1 | 2.578 | 0.6446 | Uncharacterized protein ykuL | O31698 |
| 1vac_A_cavity_1 | 3.219 | 0.6437 | H-2 class I histocompatibility antigen, K-B alpha chain | P01901 |
| 1n8z_C_cavity_2 | 2.574 | 0.6434 | Receptor tyrosine-protein kinase erbB-2 | ERBB2_HUMAN |
| 1yqt_A_cavity_5 | 2.561 | 0.6402 | RNase l inhibitor | Q8U306 |
| 2p0r_B_cavity_2 | 3.173 | 0.6346 | Calpain-9 | CAN9_HUMAN |
| 2rm4_A_cavity_3 | 3.172 | 0.6343 | Enhancer of mRNA-decapping protein 3 | Q9VVI2 |
| 2i7x_A_cavity_4 | 2.536 | 0.634 | Cleavage and polyadenylation specificity factor subunit 3 | CPSF3_HUMAN |
| 2ayn_C_cavity_2 | 3.151 | 0.6301 | Ubiquitin carboxyl-terminal hydrolase 14 | UBP14_HUMAN |
| 2i9x_A_cavity_1 | 2.478 | 0.6195 | Putative septation protein spoVG | Q8CML1 |
| 1hm9_A_cavity_4 | 3.093 | 0.6186 | Bifunctional protein glmU | Q97R46 |
| 2zv8_A_cavity_2 | 6.775 | 0.6159 | Tyrosine-protein kinase Lyn | P25911 |
| 2dba_A_cavity_1 | 2.459 | 0.6148 | Protein unc-45 homolog A | Q9H3U1 |
| 1oxy_A_cavity_2 | 3.05 | 0.61 | Hemocyanin II | P04253 |
| 1yiv_A_cavity_1 | 4.869 | 0.6086 | Myelin P2 protein | P0C6G6 |
| 1ojm_A_cavity_2 | 2.42 | 0.605 | Hyaluronate lyase | Q54873 |
| 1jfi_B_cavity_2 | 2.41 | 0.6025 | Dr1-associated corepressor | Q14919 |
| 1gm6_A_cavity_1 | 3 | 0.6 | Salivary lipocalin | P81608 |
| 2yww_B_cavity_1 | 2.986 | 0.5971 | Aspartate carbamoyltransferase regulatory chain | Q58801 |
| 2b6e_H_cavity_1 | 2.37 | 0.5926 | Putative esterase HI1161 | P45083 |
| 1vmk_B_cavity_1 | 2.954 | 0.5908 | Purine nucleoside phosphorylase | Q9X1T2 |
| 3c2o_A_cavity_1 | 2.954 | 0.5908 | Nicotinate-nucleotide pyrophosphorylase [carboxylating] | P43619 |
| 1so0_C_cavity_2 | 2.939 | 0.5878 | Aldose 1-epimerase | Q96C23 |
| 1fcx_A_cavity_3 | 2.928 | 0.5856 | Retinoic acid receptor gamma | RARG_HUMAN |
| 1cpc_B_cavity_1 | 2.923 | 0.5845 | C-phycocyanin-1 alpha chain | P07122 |
| 1zp2_A_cavity_4 | 3.484 | 0.5806 | RNA polymerase II holoenzyme cyclin-like subunit | O94503 |
| 1ukf_A_cavity_1 | 3.447 | 0.5745 | Cysteine protease avirulence protein avrPphB | Q52430 |
| 1gtt_C_cavity_5 | 2.283 | 0.5709 | Homoprotocatechuate catabolism bifunctional isomerase/decarboxylase | P37352 |
| 2fjk_C_cavity_1 | 2.278 | 0.5694 | Fructose-bisphosphate aldolase | Q703I2 |
| 1ez4_B_cavity_2 | 2.261 | 0.5653 | L-lactate dehydrogenase | P56511 |
| 3exa_A_cavity_2 | 2.821 | 0.5642 | tRNA Delta(2)-isopentenylpyrophosphate transferase | Q9KAC3 |
| 3ca8_A_cavity_1 | 2.244 | 0.5609 | Protein ydcF | P34209 |
| 1wh0_A_cavity_1 | 3.359 | 0.5599 | Ubiquitin carboxyl-terminal hydrolase 19 | UBP19_HUMAN |
| 1w98_B_cavity_1 | 3.337 | 0.5562 | G1/S-specific cyclin-E1 | CCNE1_HUMAN |
| 1poc_A_cavity_1 | 3.308 | 0.5513 | Phospholipase A2 | P00630 |
| 1mg6_A_cavity_1 | 2.732 | 0.5463 | Phospholipase A2 homolog acutohaemolysin | O57385 |
| 2dnk_A_cavity_1 | 3.277 | 0.5462 | CUG-BP- and ETR-3-like factor 4 | Q9BZC1 |
| 1ew3_A_cavity_1 | 2.185 | 0.5462 | Major allergen Equ c 1 | Q95182 |
| 2e7u_A_cavity_2 | 2.728 | 0.5455 | Glutamate-1-semialdehyde 2,1-aminomutase | Q5SJS4 |
| 1s40_A_cavity_2 | 3.271 | 0.5451 | Cell division control protein 13 | P32797 |
| 1y88_A_cavity_1 | 2.18 | 0.545 | Uncharacterized protein AF_1548 | O28724 |
| 1dus_A_cavity_2 | 3.241 | 0.5401 | Protein MJ0882 | Q58292 |
| 2qp4_A_cavity_3 | 2.16 | 0.5399 | Bile salt sulfotransferase | Q06520 |
| 2qc3_A_cavity_1 | 4.858 | 0.5398 | Malonyl CoA-acyl carrier protein transacylase | P63458 |
| 1wp1_B_cavity_1 | 2.667 | 0.5335 | Outer membrane protein oprM | OPRM_PSEAE |
| 2uzf_A_cavity_2 | 3.198 | 0.5331 | Naphthoate synthase | Q5HH38 |
| 3bkb_A_cavity_3 | 3.192 | 0.532 | Proto-oncogene tyrosine-protein kinase Fes/Fps | FES_HUMAN |
| 1gzq_A_cavity_1 | 2.654 | 0.5309 | T-cell surface glycoprotein CD1b | CD1B_HUMAN |
| 3c7g_A_cavity_3 | 3.184 | 0.5307 | Arabinoxylan arabinofuranohydrolase | Q45071 |
| 2h08_A_cavity_2 | 3.168 | 0.528 | Ribose-phosphate pyrophosphokinase 1 | P60891 |
| 1gk9_B_cavity_7 | 3.668 | 0.524 | Penicillin G acylase | P06875 |
| 1wxr_A_cavity_2 | 4.19 | 0.5238 | Hemoglobin-binding protease hbp | O88093 |
| 1fft_A_cavity_4 | 2.604 | 0.5208 | Ubiquinol oxidase subunit 1 | P0ABI8 |
| 1igw_B_cavity_1 | 3.119 | 0.5199 | Isocitrate lyase | P0A9G6 |
| 1odh_A_cavity_1 | 2.591 | 0.5182 | Chorion-specific transcription factor GCMa | P70348 |
| 2fxt_A_cavity_1 | 3.082 | 0.5136 | Mitochondrial import inner membrane translocase subunit TIM44 | Q01852 |
| 1hyh_C_cavity_1 | 3.07 | 0.5117 | L-2-hydroxyisocaproate dehydrogenase | P14295 |
| 1tno_J_cavity_3 | 3.051 | 0.5085 | Protein farnesyltransferase/geranylgeranyltransferase type-1 subunit alpha | Q04631 |
| 2pff_A_cavity_9 | 3.039 | 0.5066 | Fatty acid synthase subunit alpha | P19097 |
| 2ppi_A_cavity_1 | 3.03 | 0.505 | Gigaxonin | Q9H2C0 |
| 1a05_A_cavity_3 | 2 | 0.5 | 3-isopropylmalate dehydrogenase | Q56268 |
| 1ws1_A_cavity_1 | 2.986 | 0.4977 | Peptide deformylase 1 | Q819U0 |
| 1k8g_B_cavity_2 | 2.98 | 0.4967 | Telomere-binding protein subunit alpha | P29549 |
| 2pby_D_cavity_1 | 2.976 | 0.496 | Glutaminase | Q5KY26 |
| 1y64_B_cavity_2 | 3.964 | 0.4955 | Actin, alpha skeletal muscle | P68135 |
| 1ofh_B_cavity_1 | 2.97 | 0.495 | ATP-dependent hsl protease ATP-binding subunit hslU | P43773 |
| 1h4j_A_cavity_1 | 2.966 | 0.4943 | Methanol dehydrogenase subunit 1 | P16027 |
| 2dk2_A_cavity_2 | 2.472 | 0.4943 | Heterogeneous nuclear ribonucleoprotein R | O43390 |
| 2cxq_A_cavity_2 | 3.434 | 0.4906 | Glucose-6-phosphate isomerase | P06745 |
| 3fku_T_cavity_1 | 2.943 | 0.4906 | Hemagglutinin | Q6J8F6 |
| 1s99_B_cavity_2 | 2.451 | 0.4902 | Putative HMP/thiamine-binding protein ykoF | O34911 |
| 2kdf_A_cavity_1 | 2.939 | 0.4898 | 26S proteasome non-ATPase regulatory subunit 4 | P55036 |
| 1ma1_A_cavity_1 | 2.933 | 0.4889 | Superoxide dismutase [Fe] | P18868 |
| 1chm_A_cavity_1 | 2.933 | 0.4889 | Creatinase | P38488 |
| 2ija_A_cavity_1 | 2.927 | 0.4878 | Arylamine N-acetyltransferase 1 | ARY1_HUMAN |
| 2zuy_A_cavity_4 | 2.922 | 0.4871 | Rhamnogalacturonan lyase yesX | O31527 |
| 1rp8_A_cavity_1 | 3.895 | 0.4869 | Alpha-amylase type A isozyme | P00693 |
| 1lwu_E_cavity_2 | 2.919 | 0.4865 | Fibrinogen alpha-1 chain | P02674 |
| 1gq2_I_cavity_2 | 2.91 | 0.485 | NADP-dependent malic enzyme | P40927 |
| 1em2_A_cavity_1 | 2.42 | 0.484 | StAR-related lipid transfer protein 3 | Q14849 |
| 2z3x_A_cavity_1 | 2.88 | 0.4799 | Small, acid-soluble spore protein C | P02958 |
| 2d2z_C_cavity_1 | 2.871 | 0.4785 | Chloride intracellular channel protein 4 | Q9Y696 |
| 2ysw_C_cavity_1 | 3.824 | 0.4781 | 3-dehydroquinate dehydratase | O66440 |
| 1u3r_B_cavity_1 | 3.339 | 0.4771 | Estrogen receptor beta | ESR2_HUMAN |
| 1rso_A_cavity_1 | 2.863 | 0.4771 | Disks large homolog 1 | Q62696 |
| 2cxi_B_cavity_1 | 2.858 | 0.4764 | Phenylalanyl-tRNA synthetase beta chain | O73984 |
| 2j47_A_cavity_1 | 3.327 | 0.4753 | O-GlcNAcase BT_4395 | Q89ZI2 |
| 3bky_H_cavity_1 | 2.836 | 0.4727 | B-lymphocyte antigen CD20 | P11836 |
| 1sgm_B_cavity_1 | 2.831 | 0.4718 | Uncharacterized HTH-type transcriptional regulator yxaF | P42105 |
| 3gos_B_cavity_1 | 2.828 | 0.4714 | 2,3,4,5-tetrahydropyridine-2,6-dicarboxylate N-succinyltransferase | Q8ZH69 |
| 1nm3_A_cavity_2 | 2.34 | 0.4681 | Hybrid peroxiredoxin hyPrx5 | P44758 |
| 1a8p_A_cavity_1 | 3.274 | 0.4678 | Ferredoxin--NADP reductase | Q44532 |
| 2z7x_B_cavity_1 | 2.805 | 0.4675 | Toll-like receptor 1 | TLR1_HUMAN |
| 2c2c_A_cavity_1 | 4.203 | 0.467 | Cytochrome c2 | P0C189 |
| 1spu_B_cavity_8 | 2.801 | 0.4668 | Primary amine oxidase | P46883 |
| 2apo_A_cavity_1 | 2.801 | 0.4668 | Probable tRNA pseudouridine synthase B | Q57612 |
| 1wru_A_cavity_3 | 2.798 | 0.4663 | Baseplate protein | P08558 |
| 2vdg_A_cavity_1 | 3.261 | 0.4659 | Aldose reductase | P23901 |
| 1dw3_C_cavity_1 | 3.254 | 0.4648 | Cytochrome c-type protein SHP | P81238 |
| 3bdf_B_cavity_1 | 2.786 | 0.4644 | Alkaline phosphatase | P00634 |
| 1x86_A_cavity_2 | 2.778 | 0.4631 | Rho guanine nucleotide exchange factor 12 | ARHGC_HUMAN |
| 1ddm_A_cavity_1 | 2.305 | 0.4611 | Protein numb | P16554 |
| 2hzb_A_cavity_1 | 6.438 | 0.4599 | UPF0052 protein BH3568 | Q9K706 |
| 2owy_A_cavity_1 | 2.758 | 0.4596 | Recombination-associated protein rdgC | Q9HYX7 |
| 2bsk_D_cavity_1 | 3.676 | 0.4595 | Mitochondrial import inner membrane translocase subunit Tim9 | Q9Y5J7 |
| 1y7e_A_cavity_1 | 2.751 | 0.4585 | Probable M18 family aminopeptidase 1 | P0C925 |
| 1z9f_A_cavity_1 | 2.75 | 0.4583 | Single-stranded DNA-binding protein | Q9WZ73 |
| 1cfp_A_cavity_1 | 2.749 | 0.4581 | Protein S100-B | P02638 |
| 2zat_A_cavity_2 | 2.745 | 0.4575 | Dehydrogenase/reductase SDR family member 4 | Q8WNV7 |
| 2p0t_A_cavity_1 | 2.282 | 0.4565 | UPF0307 protein PSPTO_4464 | Q87WS9 |
| 1xpk_C_cavity_1 | 3.191 | 0.4559 | 3-hydroxy-3-methylglutaryl CoA synthase | Q79ZY6 |
| 1p9p_A_cavity_1 | 3.64 | 0.4551 | tRNA | P0A876 |
| 2arp_F_cavity_1 | 2.728 | 0.4547 | Inhibin beta A chain | P08476 |
| 3b68_A_cavity_1 | 3.181 | 0.4545 | Androgen receptor | ANDR_HUMAN |
| 2v0x_B_cavity_2 | 4.081 | 0.4534 | Lamina-associated polypeptide 2, isoforms alpha/zeta | Q61033 |
| 1vlu_B_cavity_1 | 3.163 | 0.4519 | Gamma-glutamyl phosphate reductase | P54885 |
| 1n35_A_cavity_5 | 2.244 | 0.4489 | RNA-directed RNA polymerase lambda-3 | P17378 |
| 2eb1_A_cavity_1 | 2.693 | 0.4488 | Endoribonuclease Dicer | Q9UPY3 |
| 1q8b_A_cavity_1 | 3.139 | 0.4484 | Uncharacterized protein yjcS | O31641 |
| 1w60_B_cavity_1 | 2.689 | 0.4482 | Proliferating cell nuclear antigen | PCNA_HUMAN |
| 1kvd_B_cavity_1 | 2.661 | 0.4436 | Salt-mediated killer protoxin 1 | P19972 |
| 2ee4_A_cavity_1 | 3.546 | 0.4433 | Rho GTPase-activating protein 5 | Q13017 |
| 2raj_A_cavity_3 | 2.655 | 0.4426 | Sorting nexin-9 | Q9Y5X1 |
| 1yf6_L_cavity_3 | 2.649 | 0.4414 | Reaction center protein H chain | P0C0Y7 |
| 1fiz_A_cavity_1 | 3.088 | 0.4412 | Acrosin | P08001 |
| 1trb_A_cavity_1 | 2.204 | 0.4409 | Thioredoxin reductase | P0A9P4 |
| 1kmh_B_cavity_1 | 2.642 | 0.4403 | ATP synthase subunit alpha, chloroplastic | P06450 |
| 1n8w_A_cavity_3 | 3.075 | 0.4393 | Malate synthase G | P0A5J4 |
| 1wjp_A_cavity_1 | 3.068 | 0.4383 | Zinc finger protein 295 | Q9ULJ3 |
| 2hps_A_cavity_1 | 3.498 | 0.4372 | Luciferin-binding protein | P05938 |
| 2h00_C_cavity_1 | 3.052 | 0.4361 | Putative methyltransferase METT10D | Q86W50 |
| 1m1b_B_cavity_1 | 3.051 | 0.4359 | Phosphoenolpyruvate phosphomutase | P56839 |
| 1vmi_A_cavity_2 | 3.043 | 0.4347 | Ethanolamine utilization protein eutD | P77218 |
| 2ols_A_cavity_2 | 2.605 | 0.4341 | Phosphoenolpyruvate synthase | Q9K0I2 |
| 1pvn_B_cavity_2 | 3.036 | 0.4336 | Inosine-5-monophosphate dehydrogenase | P50097 |
| 1vcn_A_cavity_1 | 3.455 | 0.4319 | CTP synthase | Q5SIA8 |
| 1ux6_A_cavity_1 | 3.454 | 0.4318 | Thrombospondin-1 | TSP1_HUMAN |
| 2be1_B_cavity_1 | 3.45 | 0.4312 | Serine/threonine-protein kinase/endoribonuclease IRE1 | P32361 |
| 3bze_A_cavity_1 | 4.308 | 0.4308 | HLA class I histocompatibility antigen, alpha chain E | HLAE_HUMAN |
| 2z2n_A_cavity_2 | 3.013 | 0.4305 | Virginiamycin B lyase | P17978 |
| 1lxt_B_cavity_2 | 3.439 | 0.4299 | Phosphoglucomutase-1 | P00949 |
| 2vwe_L_cavity_2 | 3 | 0.4286 | Vascular endothelial growth factor B | P49765 |
| 1wex_A_cavity_1 | 2.142 | 0.4284 | Heterogeneous nuclear ribonucleoprotein L-like | Q921F4 |
| 1tu9_A_cavity_1 | 2.567 | 0.4278 | Hypothetical protein | Q9HX49 |
| 1sr8_A_cavity_2 | 3.405 | 0.4257 | Putative cobalt-precorrin-6A synthase [deacetylating] | O29535 |
| 1ya0_B_cavity_2 | 2.98 | 0.4257 | Protein SMG7 | Q92540 |
| 2qlu_A_cavity_2 | 3.822 | 0.4246 | Activin receptor type-2B | Q13705 |
| 2qnc_B_cavity_1 | 2.966 | 0.4237 | Recombination endonuclease VII | P13340 |
| 1ypf_B_cavity_2 | 2.119 | 0.4237 | GMP reductase | Q81JJ9 |
| 2a5h_C_cavity_2 | 2.54 | 0.4234 | L-lysine 2,3-aminomutase | Q9XBQ8 |
| 2acm_B_cavity_1 | 2.963 | 0.4233 | Mucin-1 | P15941 |
| 1wz8_D_cavity_3 | 2.961 | 0.423 | Enoyl-CoA hydratase | Q5SLS5 |
| 1a57_A_cavity_1 | 3.806 | 0.4229 | Fatty acid-binding protein, intestinal | P02693 |
| 1ll0_A_cavity_1 | 2.958 | 0.4226 | Glycogenin-1 | P13280 |
| 1o68_A_cavity_1 | 3.798 | 0.4221 | 3-methyl-2-oxobutanoate hydroxymethyltransferase | Q9JZW6 |
| 1rym_A_cavity_1 | 3.371 | 0.4214 | Chloroplastic group IIB intron splicing facilitator CRS2, chloroplastic | Q9M5P4 |
| 2r6w_A_cavity_1 | 3.369 | 0.4211 | Estrogen receptor | ESR1_HUMAN |
| 1go3_N_cavity_1 | 3.368 | 0.4211 | DNA-directed RNA polymerase subunit E | Q57840 |
| 3c5k_A_cavity_1 | 2.94 | 0.42 | Histone deacetylase 6 | HDAC6_HUMAN |
| 1pkq_G_cavity_1 | 2.939 | 0.4199 | Myelin-oligodendrocyte glycoprotein | Q63345 |
| 3h7f_B_cavity_3 | 2.937 | 0.4195 | Serine hydroxymethyltransferase 1 | O53441 |
| 1lmz_A_cavity_1 | 2.929 | 0.4184 | DNA-3-methyladenine glycosylase 1 | P05100 |
| 3gyr_C_cavity_4 | 3.344 | 0.4179 | Phenoxazinone synthase | Q53692 |
| 3cb5_A_cavity_4 | 2.92 | 0.4172 | FACT complex subunit spt16 | O94267 |
| 1ud1_C_cavity_2 | 2.917 | 0.4167 | Glycinin G1 | P04776 |
| 2j8w_A_cavity_1 | 3.746 | 0.4162 | Cytochrome c | P00142 |
| 1bqb_A_cavity_1 | 3.329 | 0.4161 | Zinc metalloproteinase aureolysin | P81177 |
| 2zs0_C_cavity_1 | 3.309 | 0.4136 | Extracellular giant hemoglobin major globin subunit A1 | Q7M419 |
| 1d7c_A_cavity_1 | 2.475 | 0.4126 | Cellobiose dehydrogenase | Q01738 |
| 1h21_C_cavity_2 | 3.3 | 0.4125 | Split-Soret cytochrome c | P81040 |
| 3epy_B_cavity_3 | 2.887 | 0.4124 | Acyl-CoA-binding domain-containing protein 7 | Q8N6N7 |
| 1s9c_F_cavity_4 | 2.886 | 0.4124 | Peroxisomal multifunctional enzyme type 2 | P51659 |
| 1lbm_A_cavity_1 | 2.056 | 0.4112 | N-(5-phosphoribosyl)anthranilate isomerase | Q56320 |
| 3brp_A_cavity_1 | 2.877 | 0.411 | C-phycocyanin alpha chain | P00306 |
| 2egw_B_cavity_1 | 2.449 | 0.4082 | Ribosomal RNA small subunit methyltransferase E | O66552 |
| 3bvo_B_cavity_1 | 3.672 | 0.408 | Co-chaperone protein HscB, mitochondrial | Q8IWL3 |
| 2if2_C_cavity_2 | 2.448 | 0.408 | Dephospho-CoA kinase | O67792 |
| 2c62_A_cavity_1 | 2.854 | 0.4077 | Activated RNA polymerase II transcriptional coactivator p15 | P53999 |
| 2vv5_F_cavity_2 | 2.853 | 0.4076 | Small-conductance mechanosensitive channel | MSCS_ECOLI |
| 1spj_A_cavity_1 | 3.26 | 0.4075 | Kallikrein-1 | KLK1_HUMAN |
| 1b35_A_cavity_1 | 2.442 | 0.4071 | Genome polyprotein | P13418 |
| 1sjw_A_cavity_1 | 3.253 | 0.4066 | Nogalonic acid methyl ester cyclase | Q9RN59 |
| 2r6z_B_cavity_1 | 4.468 | 0.4062 | UPF0341 protein in rsp 3region | P72077 |
| 3fvm_B_cavity_3 | 3.243 | 0.4054 | Mannonate dehydratase | A4VVI4 |
| 2e74_A_cavity_3 | 2.838 | 0.4054 | Cytochrome b6 | P83791 |
| 1tw2_B_cavity_3 | 2.837 | 0.4053 | Carminomycin 4-O-methyltransferase | Q06528 |
| 2fv4_B_cavity_1 | 2.027 | 0.4053 | Kinetochore protein SPC25 | P40014 |
| 1v9x_A_cavity_1 | 5.267 | 0.4052 | Poly [ADP-ribose] polymerase 1 | Q9ZP54 |
| 2rgv_A_cavity_1 | 6.864 | 0.4038 | Peroxide operon regulator | P71086 |
| 1v1f_A_cavity_1 | 2.42 | 0.4034 | Calcineurin B-like protein 4 | O81223 |
| 1mg5_B_cavity_1 | 3.222 | 0.4027 | Alcohol dehydrogenase | P00334 |
| 1h6n_A_cavity_5 | 4.831 | 0.4025 | Catalase | P42321 |
| 2dgu_A_cavity_2 | 2.01 | 0.4019 | Heterogeneous nuclear ribonucleoprotein Q | O60506 |
| 1rkh_A_cavity_1 | 4.015 | 0.4015 | Vitamin D3 receptor | P13053 |
| 2pph_A_cavity_1 | 3.61 | 0.4011 | Mitogen-activated protein kinase kinase kinase 3 | M3K3_HUMAN |
| 2q3m_A_cavity_1 | 2.407 | 0.4011 | Flavonol sulfotransferase-like | P52839 |
| 1vmd_B_cavity_1 | 2.801 | 0.4001 | Methylglyoxal synthase | Q9X0R7 |
| 2aug_B_cavity_1 | 2 | 0.4 | Growth factor receptor-bound protein 14 | Q14449 |
| 1qcb_F_cavity_1 | 2.399 | 0.3998 | Heat-labile enterotoxin IIB, B chain | P43529 |
| 3cb4_F_cavity_2 | 4.775 | 0.3979 | GTP-binding protein lepA | P60785 |
| 1c0l_A_cavity_2 | 2.785 | 0.3979 | D-amino-acid oxidase | P80324 |
| 1jnd_A_cavity_1 | 4.768 | 0.3974 | Chitinase-like protein Idgf2 | Q9V3D4 |
| 1emy_A_cavity_1 | 3.974 | 0.3974 | Myoglobin | P02186 |
| 2rhs_B_cavity_5 | 3.177 | 0.3971 | Phenylalanyl-tRNA synthetase alpha chain | Q4L5E3 |
| 2kd2_A_cavity_1 | 2.769 | 0.3956 | Fas apoptotic inhibitory molecule 1 | Q9WUD8 |
| 1f0x_A_cavity_1 | 2.768 | 0.3955 | D-lactate dehydrogenase | DLD_ECOLI |
| 2daf_A_cavity_2 | 2.76 | 0.3943 | IQ and ubiquitin-like domain-containing protein | Q8NA54 |
| 1l3w_A_cavity_1 | 3.148 | 0.3935 | EP-cadherin | P33148 |
| 1zde_A_cavity_1 | 2.754 | 0.3935 | DNA polymerase III subunit alpha | P74750 |
| 1jqx_A_cavity_1 | 2.742 | 0.3916 | Dihydroorotate dehydrogenase A | Q53ZE5 |
| 3dpi_A_cavity_3 | 2.349 | 0.3915 | NH(3)-dependent NAD(+) synthetase | Q3JL79 |
| 3fsl_F_cavity_2 | 3.521 | 0.3912 | Aromatic-amino-acid aminotransferase | P04693 |
| 1vrb_B_cavity_3 | 2.347 | 0.3912 | Uncharacterized protein yxbC | P46327 |
| 1skq_B_cavity_6 | 3.125 | 0.3906 | Elongation factor 1-alpha | P35021 |
| 1ch4_A_cavity_1 | 3.504 | 0.3894 | Hemoglobin subunit beta | HBB_HUMAN |
| 1n83_A_cavity_1 | 3.495 | 0.3884 | Nuclear receptor ROR-alpha | RORA_HUMAN |
| 1p16_B_cavity_4 | 2.719 | 0.3884 | mRNA-capping enzyme subunit alpha | P78587 |
| 1knx_D_cavity_1 | 2.328 | 0.3881 | HPr kinase/phosphorylase | P75548 |
| 2p8u_A_cavity_3 | 3.486 | 0.3874 | Hydroxymethylglutaryl-CoA synthase, cytoplasmic | HMCS1_HUMAN |
| 1ns6_A_cavity_1 | 3.099 | 0.3873 | Hemoglobin subunit alpha | P01958 |
| 1ouu_B_cavity_1 | 3.098 | 0.3873 | Hemoglobin subunit alpha-1 | P02019 |
| 1fnc_A_cavity_1 | 3.097 | 0.3872 | Ferredoxin--NADP reductase, chloroplastic | P00455 |
| 2lh5_A_cavity_1 | 3.094 | 0.3867 | Leghemoglobin-2 | P02240 |
| 1t13_A_cavity_1 | 3.467 | 0.3853 | 6,7-dimethyl-8-ribityllumazine synthase 2 | P61713 |
| 2o3a_B_cavity_1 | 3.082 | 0.3853 | tRNA ribose 2-O-methyltransferase aTrm56 | O29507 |
| 1gup_A_cavity_2 | 2.311 | 0.3852 | Galactose-1-phosphate uridylyltransferase | P09148 |
| 2bjh_B_cavity_2 | 2.693 | 0.3848 | Feruloyl esterase A | O42807 |
| 3b9p_A_cavity_1 | 3.078 | 0.3847 | Spastin | Q8I0P1 |
| 2zsi_A_cavity_1 | 3.075 | 0.3844 | Probable gibberellin receptor GID1L1 | Q9MAA7 |
| 2do8_A_cavity_2 | 3.457 | 0.3841 | UPF0301 protein HD_1794 | Q7VKS7 |
| 1tg6_A_cavity_2 | 2.305 | 0.3841 | Putative ATP-dependent Clp protease proteolytic subunit, mitochondrial | CLPP_HUMAN |
| 3c10_C_cavity_1 | 3.072 | 0.384 | Histone deacetylase 7 | HDAC7_HUMAN |
| 1f0n_A_cavity_1 | 3.453 | 0.3837 | Antigen 85-B | P0C5B9 |
| 1f6t_B_cavity_1 | 3.068 | 0.3835 | Nucleoside diphosphate kinase, cytosolic | P22887 |
| 2nq5_A_cavity_3 | 3.448 | 0.3831 | 5-methyltetrahydropteroyltriglutamate--homocysteine methyltransferase | Q8CWX6 |
| 1x5p_A_cavity_1 | 3.064 | 0.383 | Negative elongation factor E | P18615 |
| 1hbp_A_cavity_1 | 2.296 | 0.3826 | Retinol-binding protein 4 | P18902 |
| 3prc_M_cavity_6 | 2.676 | 0.3823 | Photosynthetic reaction center cytochrome c subunit | P07173 |
| 1xp8_A_cavity_1 | 3.045 | 0.3807 | Protein recA | P42443 |
| 1nox_A_cavity_1 | 3.043 | 0.3803 | NADH dehydrogenase | Q60049 |
| 1h29_D_cavity_3 | 3.419 | 0.3799 | High-molecular-weight cytochrome c | P24092 |
| 1ef1_D_cavity_1 | 3.414 | 0.3793 | Moesin | P26038 |
| 3bw7_A_cavity_2 | 3.4 | 0.3778 | Cytokinin dehydrogenase 1 | Q9T0N8 |
| 1iq3_A_cavity_2 | 3.4 | 0.3778 | RalBP1-associated Eps domain-containing protein 2 | Q8NFH8 |
| 1l3g_A_cavity_1 | 2.266 | 0.3776 | Transcription factor MBP1 | P39678 |
| 2fk4_A_cavity_1 | 3.015 | 0.3769 | Protein E6 | P03126 |
| 1uvx_A_cavity_1 | 2.638 | 0.3769 | Globin LI637 | Q08753 |
| 2ywc_A_cavity_4 | 3.011 | 0.3764 | GMP synthase [glutamine-hydrolyzing] | Q5SI28 |
| 1vbg_A_cavity_7 | 3.001 | 0.3752 | Pyruvate, phosphate dikinase 1, chloroplastic | P11155 |
| 1dvh_A_cavity_1 | 3.373 | 0.3748 | Cytochrome c-553 | P04032 |
| 1lqj_B_cavity_1 | 2.247 | 0.3745 | Uracil-DNA glycosylase | P12295 |
| 1bip_A_cavity_3 | 2.239 | 0.3732 | Alpha-amylase/trypsin inhibitor | P01087 |
| 1fmb_A_cavity_1 | 2.977 | 0.3721 | Pol polyprotein | P32542 |
| 1i1m_A_cavity_1 | 2.972 | 0.3715 | Branched-chain-amino-acid aminotransferase | P0AB80 |
| 1w9y_A_cavity_1 | 3.338 | 0.3708 | 1-aminocyclopropane-1-carboxylate oxidase 1 | Q08506 |
| 1vim_B_cavity_1 | 2.967 | 0.3708 | Hypothetical protein AF_1796 | O28478 |
| 2qvv_A_cavity_1 | 2.593 | 0.3704 | Fructose-1,6-bisphosphatase 1 | P00636 |
| 2b7m_A_cavity_1 | 2.962 | 0.3703 | Exocyst complex component EXO70 | P19658 |
| 1e77_A_cavity_3 | 2.591 | 0.3702 | Glucose-6-phosphate 1-dehydrogenase | P11411 |
| 2qgh_A_cavity_1 | 3.331 | 0.3701 | Diaminopimelate decarboxylase | P56129 |
| 2ex8_A_cavity_3 | 2.221 | 0.3701 | D-alanyl-D-alanine carboxypeptidase dacB | P24228 |
| 2zfh_B_cavity_1 | 2.959 | 0.3699 | Protein CutA | O60888 |
| 3din_C_cavity_7 | 2.954 | 0.3692 | Protein translocase subunit secA | Q9X1R4 |
| 2dy3_C_cavity_1 | 3.321 | 0.369 | Alanine racemase | Q8RSU9 |
| 2e3x_A_cavity_5 | 2.952 | 0.369 | Coagulation factor X-activating enzyme heavy chain | Q7LZ61 |
| 2jqe_A_cavity_2 | 3.32 | 0.3689 | Signal recognition 54 kDa protein | O29633 |
| 2tpr_B_cavity_4 | 2.212 | 0.3687 | Trypanothione reductase | P39040 |
| 2igi_B_cavity_1 | 2.947 | 0.3684 | Oligoribonuclease | P0A784 |
| 2vwt_A_cavity_1 | 3.315 | 0.3683 | 2-keto-3-deoxy-L-rhamnonate aldolase | P76469 |
| 1b0n_B_cavity_1 | 2.577 | 0.3682 | HTH-type transcriptional regulator sinR | P06533 |
| 2k27_A_cavity_1 | 2.577 | 0.3681 | Paired box protein Pax-8 | Q06710 |
| 1xc4_A_cavity_1 | 3.31 | 0.3678 | Tryptophan synthase alpha chain | P0A877 |
| 1xx6_B_cavity_1 | 3.31 | 0.3678 | Thymidine kinase | Q97F65 |
| 1u63_C_cavity_2 | 2.574 | 0.3677 | 50S ribosomal protein L1P | P54050 |
| 1d5y_D_cavity_1 | 2.94 | 0.3675 | Right origin-binding protein | P0ACI0 |
| 1sy6_H_cavity_1 | 2.938 | 0.3673 | T-cell surface glycoprotein CD3 epsilon chain | CD3E_HUMAN |
| 1x9i_A_cavity_1 | 3.303 | 0.3671 | Bifunctional phosphoglucose/phosphomannose isomerase | Q8ZWV0 |
| 1h97_B_cavity_1 | 3.298 | 0.3665 | Globin-3 | P80721 |
| 1id3_A_cavity_2 | 3.291 | 0.3657 | Histone H3 | P61830 |
| 2buw_A_cavity_2 | 3.65 | 0.365 | Protocatechuate 3,4-dioxygenase alpha chain | P20371 |
| 1qjj_A_cavity_1 | 3.283 | 0.3648 | Astacin | P07584 |
| 1smq_C_cavity_1 | 2.546 | 0.3637 | Ribonucleoside-diphosphate reductase small chain 1 | P09938 |

| Supplementary Table3: Top 10 in network string_interactions.tsv_Degree_top10 ranked by MCC method | | | |
| --- | --- | --- | --- |
| Rank | Name | Score | Encoded Protein |
| 1 | Alpp | 270 | alkaline phosphatase |
| 2 | Tnf | 264 | tumor necrosis factor |
| 2 | Ptgs2 | 264 | prostaglandin-endoperoxide synthase 2 |
| 4 | Cat | 252 | catalase |
| 5 | Nos2 | 240 | nitric oxide synthase 2 |
| 6 | Esr1 | 144 | estrogen receptor 1 |
| 7 | Gsr | 132 | glutathione-disulfide reductase |
| 8 | Cyp19a1 | 24 | cytochrome P450 family 19 subfamily A member 1 |
| 9 | G6pd | 12 | glucose-6-phosphate dehydrogenase |
| 10 | Gpi | 6 | glucose-6-phosphate isomerase |
